# Convergent domestication of bitter apples and pears by selecting mutations of MYB transcription factors to reduce proanthocyanidin levels

**DOI:** 10.1186/s43897-025-00173-z

**Published:** 2025-09-04

**Authors:** Yarong Wang, Bin Xia, Qiong Lin, Huan Wang, Zhiyong Wu, Haiqing Zhang, Zhe Zhou, Zhenli Yan, Qiming Gao, Xiangzhan Zhang, Suke Wang, Zhenzhen Liu, Xiangpeng Meng, Yaru Zhang, Andrew P. Gleave, Hengtao Zhang, Jia-Long Yao

**Affiliations:** 1 Zhengzhou Fruit Research Institute, Chinese Academy of Agricultural Sciences, 28 Gangwan Road, Zhengzhou, 450009 China; 2 The New Zealand Institute for Plant & Food Research Limited, Private Bag 92169, Auckland, 1142 New Zealand; 3 Institute of Food Science and Technology, Chinese Academy of Agricultural Sciences, Beijing, 100193 China; 4 Zhongyuan Research Center, Chinese Academy of Agricultural Sciences, Xinxiang, Henan China

**Keywords:** Domestication, Comparative genomics, *Malus*, *Pyrus*, Proanthocyanidin, Selective sweep

## Abstract

Fruit domestication has long aimed to reduce bitterness, yet the molecular mechanisms behind this trait remain only partially understood. Wild apples and pears naturally accumulate high levels of bitter proanthocyanidins (PAs), also known as condensed tannins. In this study, a convergent domestication process was identified in both fruits, involving the selection of weak alleles of MYB transcription factors that regulate PA biosynthesis. In apples, domestication targeted the *MYB-Tannin-Tamer* (*MdMYBTT*) gene. A 411-base pair transposable element inserted into the third exon of this gene in cultivated varieties produced a truncated, non-functional protein unable to activate the PA biosynthetic gene *Anthocyanidin Reductase 1* (*ANR1*). The resulting *mdmybtt* allele led to reduced PA levels and was fixed in domesticated apples through positive selection. Likewise, in pears, a 57-base pair insertion in the promoter of the *MYBPA1* gene suppressed its expression in cultivated varieties, limiting PA production. This insertion created the *mybpa1* allele, which was similarly fixed during pear domestication. These findings highlight a shared evolutionary strategy to reduce fruit bitterness by selecting mutations that suppress PA synthesis. These findings offer valuable insights into the molecular basis of domestication and inform breeding efforts to optimize both flavor and nutritional quality.

**Supplementary Information:**

The online version contains supplementary material available at 10.1186/s43897-025-00173-z.

## Core

A convergent domestication process in apples and pears targeted bitterness by selecting weak alleles of MYB transcription factors that regulate the biosynthesis of the bitter compound proanthocyanidin (PA). In apples, this involved the selection of a non-functional allele of the* MYB-Tannin-Tamer* (*MdMYBTT*) gene, which lost its ability to activate PA production. Similarly, in pears, a 57-base pair insertion in the promoter region of the *MYBPA1 *gene led to reduced gene expression in cultivated varieties, thereby limiting PA biosynthesis.

## Gene & Accession numbers

MdMYBTT accession: MD15G1051400, Pear MYBPA1 accession: Pspp.Chr07.01261.1.

## Introduction

The domestication of fruits and vegetables frequently involves the reduction of bitterness (Dar et al. 2021). In almond (*Prunus dulcis*, syn. *P. amygdalus*), bitterness results from the accumulation of amygdalin, a cyanogenic diglucoside, which is controlled by the *Sweet kernel* (*Sk*) locus. The *Sk* gene encodes a transcription factor (TF) of the basic helix–loop–helix (bHLH) family. A non-synonymous point mutation (Leu to Phe) in the bHLH inhibits transcription of the first two genes of the amygdalin biosynthetic pathway, resulting in sweet almonds (Sánchez-Pérez et al. 2019). In the Cucurbit family, which includes cucumbers, melons, and watermelons, bitterness stems from triterpenoid compounds called cucurbitacins, whose production is also regulated by a bHLH TF (*Bt*, bitter fruit). Mutations in the promoter or coding region of *Bt* eliminate fruit bitterness, and this non-bitter trait has been selected and fixed during the domestication of cucumbers, melons, and watermelons (Shang et al. 2014; Zhou et al. 2016).

Apples (*Malus domestica*) and pears (*Pyrus* spp.) represent some of the most economically and nutritionally important fruit crops worldwide. Their culinary versatility, rich nutritional content, and broad consumer appeal establish them as staples in global markets. These fruits constitute a vital component of a balanced diet, providing essential vitamins, minerals, antioxidants, and dietary fiber. Their extensive cultivation and consumption contribute substantially to agricultural economies and public health initiatives.

Domesticated apples and pears originate from wild species that produce small (< 1 cm in diameter) and bitter fruits with high levels of condensed tannins, also known as proanthocyanidins (PAs) (Cornille et al. 2014; Juniper and Mabberley 2006; Li et al. 2021; Wu et al. 2022). While a genetic variant associated with increased fruit size has been identified as a transposon insertion in the *microRNA172p* gene (Yao et al. 2015), the genetic basis for reduced bitterness in apples and pears remains unknown.

PAs present a dual challenge: although they contribute to bitterness, they also enhance nutritional value by functioning as strong antioxidants (Li et al. 2021). Hence, understanding the genetic mechanisms underlying reduced PA content is crucial to develop apple and pear cultivars with improved taste and consumer appeal and to facilitate the production of fruits with enhanced nutritional profiles.

PA is synthesized through the flavonoid pathway that also produces many other plant secondary metabolites, including flavones, flavonols, and anthocyanins (Wang et al. 2020). PA and anthocyanin biosynthesis processes share a series of enzymes, including phenylalanine ammonia lyase, chalcone synthetase, chalcone isomerase, dihydroalkanone 3-hydroxylase, dihydroflavonol-4-reductase, and anthocyanidin synthase (ANS) (Wang et al. 2020). The expression of the genes encoding these enzymes is regulated by the MBW (MYB, bHLH, and WD40) complex of TFs (Allan et al. 2008; Jaakola 2013; Wang et al. 2020). MYB proteins involved in the MBW complex are highly conserved in many plant species and are categorized into subgroup 6 of the R2R3 MYB family, which contains two conserved DNA-binding domains (Allan and Espley 2018; Allan et al. 2008). These MYBs often function as a limiting factor of the MBW complex, and their overexpression can significantly enhance red coloration in numerous plant species, including *Arabidopsis* (Borevitz et al. 2000), apple (Ding et al. 2022; Espley et al. 2009, 2007; Takos et al. 2006), and strawberry (Lin-Wang et al. 2014). In apples, two key MYB genes have been confirmed to regulate anthocyanin biosynthesis. The first gene was discovered by three different groups and named as *MdMYB1* (Takos et al. 2006), *MdMYB10* (Espley et al. 2007), and *MdMYBA* (Ban et al. 2007), representing three different alleles of the same gene (Allan and Espley 2018). A variant of *MdMYB10,* containing six small repeats in the promoter region, is important for red fruit flesh and red leaves (Espleyet al. 2009). A long terminal repeat retrotransposon insertion in the promoter region of *MdMYB10/10A* is responsible for activating gene expression in fruit skin and flower petals (Tian et al. 2022; Wang et al. 2023; Zhang et al. 2019a).

Additional enzymes, namely leucoanthocyanidin reductase (LAR) and anthocyanidin reductase (ANR), are specifically required for PA biosynthesis (Wang et al. 2020). The expression of genes encoding these enzymes is regulated by MYBs belonging to subgroups 5 and 47 (SG5 and SG47) of the R2R3 MYB family (Bogs et al. 2007; Jiao et al. 2023; Passeri et al. 2017; Primetta et al. 2015; Wang et al. 2018). In apples, the SG5 (MdMYB9, MdMYB11, and MdMYB12) and SG47 (MYBPA1) proteins positively regulate PA biosynthesis (An et al. 2018, 2015; Wang et al. 2018). In pears, PbMYB9 and PbMYB5-like MYBs regulate PA biosynthesis (Li et al. 2024a; Zhai et al. 2015). However, genetic variants of these regulatory genes significant to PA accumulation remain unknown. A genome-wide association study (GWAS) identified single nucleotide polymorphisms (SNPs) of *MdMYB9-like* (MD15G1051400) associated with PA levels in apples (Lin et al. 2023). However, the function of this gene was not fully determined, and the causal variant was also not identified. SG47 MYBs, such as VvMYBPA1 and VuMYBPA1, are directly linked to PA synthesis (Bogs et al. 2007; Passeri et al. 2017; Primetta et al. 2015; Wang et al. 2018).

The present study aimed to reveal the genetic mechanisms regulating PA levels in apples and pears and understand their roles in domestication. We first identified MYB genes of the SG5/47 within selection sweeps and then conducted comprehensive genetic analyses and functional characterization to determine specific MYB variants controlling PA levels. This discovery clarifies the genetic control of PA content and domestication of bitterness in apples and pears. By elucidating the role of these MYB proteins in PA regulation, this research contributes to the broader understanding of the genetic basis of fruit quality traits and facilitates targeted breeding and gene editing to improve fruit quality.

## Results

### *MdMYBTT* located within the selective sweep region is associated with domestication of bitter apples

**Fig. 1:**
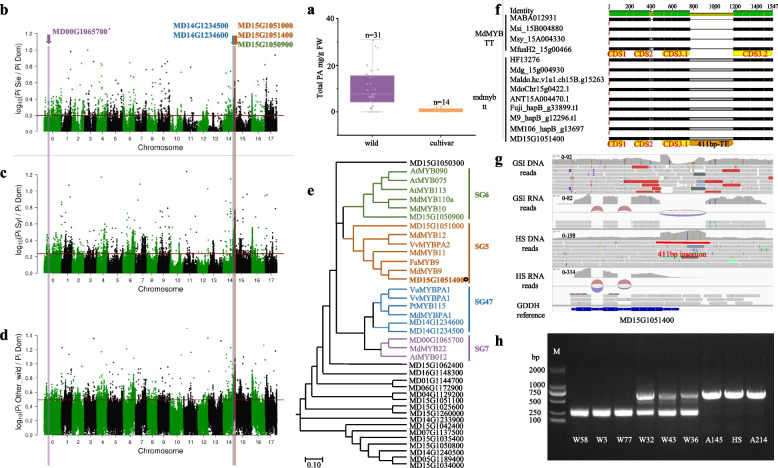
Identification of the MYB genes with a potential role in apple domestication. **a** illustrates proanthocyanidin (PA) content in mature fruits of 31 wild accessions and 14 apple cultivars. **b-d** selection sweep analysis involving comparison of *M. domestica* with *M. sieversii*, *M. sylvestris*, and other wild *Malus* accessions. The horizontal red line indicates the top 5% Pi ratio. **e** a phylogenetic tree of MYBs identified through selection sweep analyses. **f** DNA sequence alignment of MD15G1051400 and its alleles identified from different reference genomes of four wild species (*M. baccata*, *M. sieversii*, *M. sylvestris*, and *M. fusca*) and eight *M. domestica* cultivars (Hanfu, Gala, Honeycrisp, WA38, Antonovka, Fuji, M9, and MM106). **g** a snapshot of IGV view demonstrates DNA and RNA sequence reads of *M. sieboldii* (GSI) and *M. domestica* “Huashuo” (HS) mapped to the GDDH13 reference genome. **h** PCR analysis of six wild accessions and three cultivars of apples revealed two DNA bands, with the upper bands containing the 411-bp insert. W58, *M. sieversii*; W3, *M. baccata*; W77, *M. toringoides*; W32, *M. micromalus*; W43, *M. robusta*; W36, *M. prunifolia*; A145, HS, and A214 are *M. domestica*. The following GenBank or Arabidopsis TAIR accession numbers or Gene ID in GDR were used: *M. domestica MdMYB9* (MD08G1070700), *MdMYB11* (MD09G1184000), *MdMYB12* (MD15G1215500), *MdMYBPA1* (MD07G1153200), *MdMYB22* (MD03G1297100), *MdMYB10* (MD09G1278600), *MdMYB110a* (MD17G1261000); *Arabidopsis thaliana AtMYB012* (At2g47460), *AtMYB113* (At1g66370), *AtMYB075* (At1g56650), *AtMYB090* (At1g66390); *Vitis vinifera VvMYBPA2* (ACK56131), *VvMYBPA1* (CAJ90831); *Vaccinium uliginosum VuMYBPA1* (KR106180); *Fragaria ananassa FaMYB9* (AFL02460); *Populus trichocarpa* PtMYB115 (Potri.002G173900.1)

Compared to cultivated apples, wild apples normally contain significantly higher levels of PAs (Fig. 1a, Table S1) that primarily contribute to fruit bitterness (Wu et al. 2022). To identify candidate genes and causative variants for apple bitterness domestication, selective sweep analyses were conducted to identify genes with signature of adaptation, followed by functional investigations of their potential roles, consistent with previous bottom-up approaches (Ross-Ibarra et al. 2007).

As MYB TFs, particularly those belonging to SG5 and SG47, regulate PA biosynthesis (Wu et al. 2022), we first identified MYBs located within the selective sweeps between cultivated and wild apples. Based on a published dataset (Sun et al. 2020), 23 *MYB* genes were identified within the selective sweeps when *M. domestica* accessions were compared with *M. sieversii, M. sylvestris*, and other wild *Malus* accessions (Figs. 1b-d, Fig. S1, Tables S2-S4). Two *MYB*s were identified from the same selective sweep region on chromosome 15 (MD15G1051000 and MD15G1051400) and classified as subgroup SG5 (Fig. 1e). Two *MYB*s (MD14G1234500 and MD14G1234600) were classified as SG47 (Fig. 1e).

DNA variation potentially affecting gene function was identified in MD15G1051400, which contains a 411-bp insertion within the third exon (CDS3). Based on the alignment of reference genome sequences, this insertion was detected in nine *M. domestica* accessions but was absent in four wild *Malus* accessions (Fig. 1f). The insertion was identified as a transposable element (TE), flanked by an 8-base direct duplication sequence (TTCCTAGT) (Fig. S2); it showed high sequence homology to more than 90 sequences in the apple genome (Fig. S3). It also matched previously reported miniature inverted-repeat transposable elements (Chen et al. 2014). The TE insertion generated a premature stop codon and was associated with reduced transcript length (Figs. 1f-g, Fig. S2), likely resulting in a loss-of-function mutation. PCR analysis with primers flanking the insertion site effectively differentiated alleles with and without the insertion (Fig. 1h).

Among the three additional SG5 and SG47 genes identified, MD15G1051000, related to the *PH4* gene in petunia, which regulates vacuole acidification (Quattrocchio et al. 2006), did not exhibit any notable structural variations. Similarly, no remarkable structural variations were identified in MD14G1234500 and MD14G1234600 (Fig. S4). Therefore, these genes were excluded from further investigations.

Phylogenetic analysis revealed that MD15G1051400 was closely related to MYB proteins known to regulate PA biosynthesis, including MdMYB9, MdMYB11, and MdMYB12 in apples and VvMYBPA2 in grapes (Fig. 1e). Therefore, MD15G1051400 was designated *M. domestica* MYB-Tannin-Tamer (*MdMYBTT*) based on its sequence homology to PA regulators, location in the selective sweep, and potential loss-of-function mutation. The allele containing the TE insertion is designated as *mdmybtt*, while the wild-type allele without the TE insertion is termed *MdMYBTT*.

To determine the distribution of *MdMYBTT* and *mdmybtt* alleles, we genotyped 459 *Malus* accessions by PCR and whole-genome resequencing (Table 1, Table S5). Among 364 M*. domestica* accessions, 359 were homozygous *mdmybtt*, 4 were heterozygous, and only 1 was homozygous *MdMYBTT*, resulting in a *mdmybtt* allele frequency of 99.18%. Conversely, of 95 accessions from wild *Malus* species, only 5 were homozygous *mdmybtt*, indicating that the *mdmybtt* allele became fixed in *M. domestica* through selection for reduced tannin levels. The high fixation index (*Fst*) value of 0.30 further indicates that *mdmybtt* was positively selected during apple domestication.

**Table 1:** MdMYBTT genotypes in apple cultivars and wild accessions

Section	Series	Species	N	MdMYBTT	heterozygous	mdmybtt	mdmybtt allele frequency
*Malus*	*Malus*	x *domestica*	364	1	4	359	99.18%
		*sieversii*	21	6	12	3	42.86%
		*sylvestris*	4	3	1		12.50%
		*micromalus*	8	2	5	1	43.75%
		*asiatica*	2		2		50.00%
		*prunifoiia*	4	1	3		37.50%
		*floribunda*	1		1		50.00%
		*spectabilis*	7		6	1	57.14%
*Baccatus*	*Baccatae*	*baccata*	16	9	7		21.88%
		*rockii*	3	2	1		16.67%
		*mandshurica*	1	1			0
		*robusta*	8	2	6		37.50%
	*Hupehenses*	*hupehensis*	2	2			0
		*pinyiensis*	1	1			0
	*Sikkimenses*	*sikkimensis*	1	1			0
*Sorbomalus*	*Sieboldianae*	*sieboldii*	6	3	3		25.00%
		*komarovii*	2		2		50.00%
	*Kansuenses*	*kansuensis*	1	1			0
		*toringoides*	4	3	1		12.50%
		*xiaoginensis*	1	1			0
	*Yunnanensis*	*honanensis*	2	1	1		25.00%
Total			459	40	55	364	85.29%

### *mdmybtt* does not promote PA biosynthesis

**Fig. 2:**
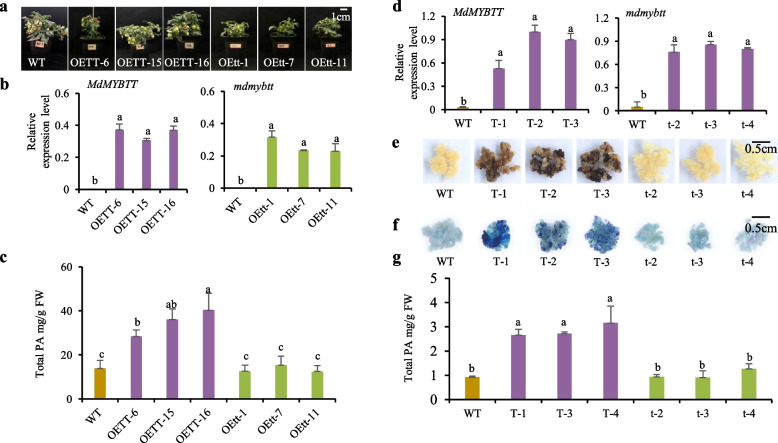
Overexpression of *MdMYBTT* and *mdmybtt* in tomato plants and apple callus. **a** photographs show one wild-type (WT) and six transgenic “Micro-Tom” tomato plants [OETT-6, OETT-15, and OETT-16 for *MdMYBTT* overexpression and OEtt-1, OEtt-7, and OEtt-11 for *mdmybtt* overexpression]. **b** relative transcript levels of *MdMYBTT* and *mdmybtt* were determined using qRT-PCR. **c** total PA levels were estimated using the HCl-vanillin method. **d** relative transcript levels of *MdMYBTT* and *mdmybtt* were assessed using qRT-PCR. **e** photographs show one WT and six transgenic callus lines of *M. domestica* “Wanglin” [T-1, T-2, and T-3 for *MdMYBTT* overexpression and t-1, t-7, and t-11 for *mdmybtt* overexpression. **f** photographs show DMACA staining of one WT and six transgenic callus lines. **g** total PA levels were quantified using the HCl-vanillin method. Error bars represent standard deviation of three biological replicates. Based on Fisher’s least significant difference (LSD) test, significant difference at the 0.05 level is indicated by different lowercase letters

The truncated protein mdmybtt retains the R2R3 DNA-binding domain but lacks the C-terminal domain (Fig. S5), which mediates protein–protein interactions (Zhao et al. 2013). To determine whether *mdmybtt* and *MdMYBTT* differ in their regulatory function in PA biosynthesis, we generated transgenic tomato plants overexpressing the *MdMYBTT* and *mdmybtt* cDNA separately (Fig. 2a, Fig. S6). The three *MdMYBTT* and three *mdmybtt* transgenic lines showed comparable levels of overexpression of the transgene RNA (Fig. 2b, Fig. S6)*.* Interestingly, only *MdMYBTT* overexpression significantly increased PA content in tomato leaves (Fig. 2c), suggesting that *MdMYBTT* plays a role in promoting PA biosynthesis, while *mdmybtt* shows loss of this function.

To validate these findings, we produced stable transgenic apple callus lines overexpressing *MdMYBTT* and *mdmybtt* cDNA. The transgene RNA exhibited similar overexpression levels in three *MdMYBTT* and three *mdmybtt* transgenic callus lines (Fig. 2d)*.* Consistent with the results in tomato plants, only *MdMYBTT* overexpression significantly increased PA content in the apple callus, evidenced by brown callus color, darker PA staining, and quantitative PA analysis (Figs. 2e-g). These findings confirmed that the *mdmybtt* allele has lost its functional role of promoting PA biosynthesis.

### MdMYBTT binds to and activates the *MdANR1* promoter

**Fig. 3:**
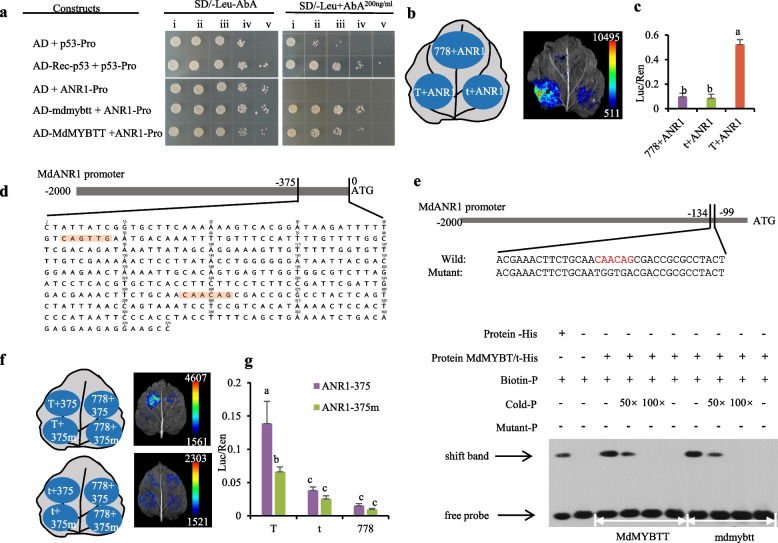
MdMYBTT binds to and activates the *ANR1* promoter. **a** yeast cells co-transformed with the constructs named on the left were cultured on SD/–Leu/– aureobasidin A (AbA^200^) non-selective medium (left panel) and SD/–Leu/ + AbA^200^ selective medium (right panel), in a dilution series of 1, 10^–1^, 10^–2^, 10^–3^, and 10^–4^ (i-v). Yeast growth on selective medium demonstrates the physical interaction between MdMYBTT and mdmybtt with the *MdANR* (MD10G1311100) promoter. **b-c** a dual-luciferase reporter assay was performed by transforming *Nicotiana benthamiana* leaves with the combinations of two plasmid constructs (b) and analyzing the Luc/Ren ratio post-transformation (c). 778, pSAK778; ANR1, *MdANR1* promoter; T, MdMYBTT, t, mdmybtt. **d** shows the 375-bp sequence upstream of *MdANR1* ATG, containing two *MYBCORE* binding motifs (highlighted in orange). **e,** EMSA shows MdMYBTT and mdmybtt binding to the *MYBCORE* cis-regulatory elements of the *MdANR1* promoter in vitro. The symbols + and – indicate the presence and absence of the corresponding proteins and probes. **f**, *N. benthamiana* leaves were co-transformed with the combinations of two plasmid constructs. 778, pSAK778; 375/375 m, pGreenII 0800-pANR1-375/375 m, T, pSAK778-MYBTT; t, pSAK778-mybtt. **g** the Luc/Ren ratio of the transformation experiments described in **f**. Error bars represent standard deviation of three biological replicates. Based on Fisher’s LSD test, significant difference at the 0.05 level is indicated by different lowercase letters

To elucidate how MdMYBTT enhances PA biosynthesis, we assessed whether MdMYBTT binds to the promoter sequence of key PA biosynthesis genes, including *ANR1, ANR2, LAR1, LAR2, ANS1*, and *ANS2*, by conducting yeast-one hybrid (Y1H) analyses. Both mdmybtt and MdMYBTT proteins exhibited strong binding to the *ANR1* promoter (Fig. 3a); however, MdMYBTT alone strongly activated the *ANR1* promoter (Figs. 3b, c). Because mdmybtt retains an intact DNA-binding domain, we expected that both mdmybtt and MdMYBTT will have similar DNA-binding ability. Additionally, the results showed no significant binding to or activation of *ANR2*, *LAR1*, *LAR2*, *ANS1*, or *ANS2* promoters by MdMYBTT (Figs. S7 a-c).

MYB proteins can bind to *MYBCORE* cis-regulatory elements (Wang et al. 2017), and two such elements were identified in the *MdANR1* promoter (Fig. 3d). The binding of MdMYBTT and mdmybtt proteins to the *MYBCORE* elements was demonstrated by electrophoretic mobility shift assay (EMSA) (Fig. 3e). MdMYBTT strongly activated the *MdANR1* wild-type (WT) promoter carrying the two *MYBCORE* elements. Although MdMYBTT activated the *MdANR1* promoter with mutated *MYBCORE* sites, the activation level was substantially reduced as compared to that for the WT promoter (Figs. 3f, g), suggesting that these *MYBCORE* elements are crucial for enhancing *MdANR1* expression through interaction with MdMYBTT and potentially other MYB proteins.

### The *mdmybtt* allele is associated with a low PA level in apple fruit

**Fig. 4:**
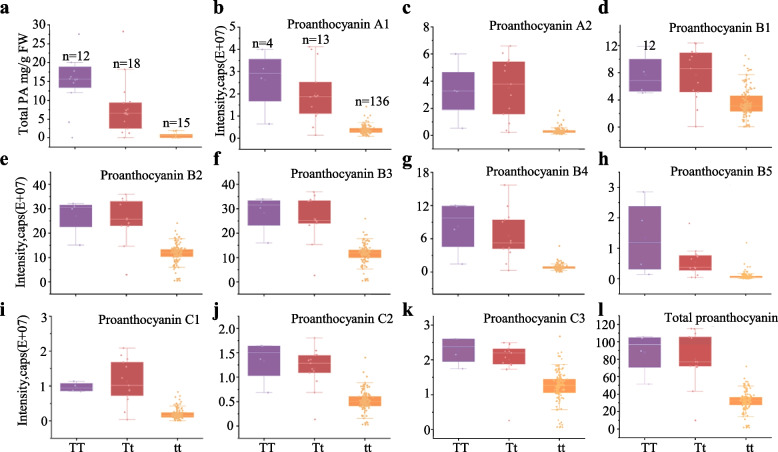
Relationship between PA content in apple fruit flesh and *MdMYBTT* genotypes. **a,** PA content in the fruit flesh of 12 *MdMYBTT* homozygous (TT), 18 heterozygous (Tt), and 15 *mdmybtt* homozygous (tt) apple accessions. **b-l** relative amount of individual (**b-k**) and total (**l**) PAs in the fruit flesh of 4 *MdMYBTT* homozygous (TT), 13 heterozygous (Tt), and 136 *mdmybtt* homozygous (tt) apple accessions

Given that MdMYBTT enhances PA biosynthesis but mdmybtt does not, we hypothesized that *Malus* accessions with *mdmybtt* alleles would accumulate lower levels of PAs than those with heterozygous or *MdMYBTT* alleles. We first genotyped the 45 *Malus* accessions previously examined for total PA content (Fig. 1a, Table S1); among these accessions, 15 were identified as *mdmybtt* homozygous, 18 as heterozygous, and 12 as *MdMYBTT* homozygous specimens. The *mdmybtt* accessions exhibited significantly lower PA levels than heterozygous and *MdMYBTT* homozygous accessions (Fig. 4a). We further extended this analysis by associating the genotyping data with PA levels in 153 *Malus* accessions from a previous study (Table S6) (Lin et al. 2023). The results revealed that 10 major PA types (PA A1, A2, B1, B2, B3, B4, B5, C1, C2, and C3) were significantly reduced in 136 *mdmybtt* homozygous accessions as compared to that in 13 heterozygous and 4 *MdMYBTT* homozygous accessions (Figs. 4b-k).

### Domestication of bitter pears through selection of a mutated MYB TF

**Fig. 5:**
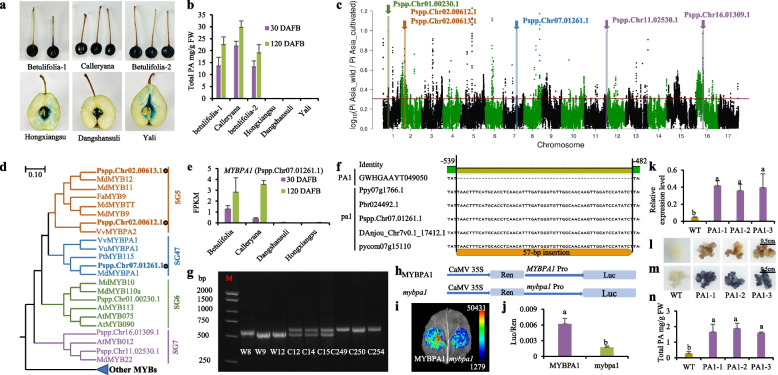
Mutation of the *MYBPA1* promotor reduces PA content in pear fruit flesh. **a** DMACA staining of pear fruit collected from three wild accessions (Betulifolia-1, Calleryana, and Betulifolia-2) and three cultivars (Hongxiangsu, Dangshansuli, and Yali) at 120 days after full bloom (DAFB). **b** PA content in the fruit flesh of the three wild accessions and three cultivars at 30 and 120 DAFB. **c,** Selective sweep analysis of 32 wild accessions and 40 cultivars of Asian pears based on Pi values. The horizontal red line indicates the top 5% Pi ratio. **d** A phylogenetic tree of the MYBs identified from the selective analysis and MYBs with known functions. **e** MYBPA1 transcript levels (FPKM) in fruit flesh collected from two wild accessions (Betulifolia and Calleryana) and two cultivars (Dangshansuli and Hongxiangsu) at 30 and 120 DAFB. **f** Alignment of the DNA sequences of *MYBPA1* downloaded from six different pear reference genomes. The gene IDs are derived from the reference genes of one wild (*P. betulifolia* “Shanxiduli”), three Asia pear cultivars (*P. pyrifolia* “Nijisseiki*,*” “Dangshansuli*,*” and “Yunhong No. 1”) and two Europe pear cultivars (*P. communis* “d’Anjou” and “Bartlett”). **g,** PCR analysis of three wild accessions and six cultivars of pear revealed two DNA bands, with the upper band containing the 57-bp insert. W8 and W9, *P. betulifolia*; W12, *P. calleryana*; C12, C14, and C15, *P. pyrifolia*; C249, C250, and C254, *P. bretschneideri*. **h-j** the promoter activities of *MYBPA1* and *mybpa1* were compared using a dual-luciferase reporter assay with two reporter constructs (**h**) transformation of *N. benthamiana* leaves (**i**), and estimation of the Luc/Ren ratio (**j**). **k** relative expression level of *MYBPA1* in one WT and three transgenic callus lines (PA1-1, PA1-2, and PA1-3) of *M. domestica* “Wanglin”. **l-m** photographs show callus of one WT and three transgenic callus lines of *M. domestica* “Wanglin.” The callus in **m** was stained with DMACA. **n** Total PA levels were determined using the HCl-vanillin method. Error bars represent standard deviation of three biological replicates. Based on Fisher’s LSD test, significant difference at the 0.05 level is indicated by different lowercase letters

Because pears (genus *Pyrus*) are closely related to apples in the Rosaceae family and produce the same type of fruit, i.e., pomes, we investigated whether they share convergent domestication patterns related to bitterness. The analysis of the total PA content in three wild and three cultivated pear varieties at 30 and 120 days after full bloom (DAFB) revealed significantly higher PA levels in wild pears than in cultivated pears (Figs. 5a, b, Table S7).

To further explore this aspect, we conducted domestication selective sweep analyses using a previously released genome sequence dataset of cultivated and wild pear accessions (Wu et al. 2018) and a high-quality reference genome of *P. pyrifolia* “Yunhong No. 1” (Sun et al. 2023a). The analysis identified two MYB SG5 genes (Pspp.Chr02.00612.1 and Pspp.Chr02.00613.1) and one MYB SG47 gene (Pspp.Chr07.01261.1, with highest homology to MdMYBPA1) in the selective sweeps between cultivated and wild Asian pears (Figs. 5c, d, Table S8) based on the top 5% Pi ratio. In contrast, a similar analysis for European pears showed absence of MYB genes belonging to SG5 and SG47 (Fig. S8, Table S9). This difference in genes under selection is consistent with the independent domestication events of Asian and European pears (Wu et al. 2018).

To identify selective sweep genes showing differential expression patterns, transcriptome analysis was conducted with RNA samples from fruit tissues of cultivated and wild Asian pears. The results showed that Pspp.Chr07.01261.1 (*MYBPA1)* transcript levels were significantly higher in wild pear accessions than in cultivated pears (Fig. 5e). This differential expression was not observed for the other two MYB genes Pspp.Chr02.00612.1 and Pspp.Chr02.00613.1 because they were not expressed in fruit tissues (Fig. S9).

Given that *MYBPA1* exhibits differential expression, resides in a selective sweep, and shares close homology with *MdMYBPA1*, which is known to positively regulate PA biosynthesis (Wang et al. 2018), we investigated genetic variants of this gene that might explain the reduced *MYBPA1* expression and PA content in cultivated pears. Sequence alignment of reference genomes from one Asian wild pear (*P. betulifolia*), three Asian cultivars (*P. pyrifolia* “Nijisseiki”, “Dangshansuli”, and “Yunhong No. 1”), and two European cultivars (*P. communis* “d’Anjou” and “Bartlett”) revealed a 57-bp insert in the *MYBPA1* promoter of cultivated pears compared to wild Asian pears (Fig. 5f). PCR analysis confirmed the presence of two alleles: *mybpa1* with the insert and *MYBPA1* without the insert (Fig. 5g). Luciferase reporter assays revealed higher promoter activity for *MYBPA1* than for *mybpa1* (Figs. 5h-j), indicating that the 57-bp insertion is associated with reduced promoter activity. Overexpression of *MYBPA1* coding sequence (CDS) in apple callus enhanced PA accumulation (Figs. 5k-m), thus confirming the role of MYBPA1 in promoting PA biosynthesis.

Further genotyping of 325 *Pyrus* accessions revealed that 210 of the 250 Asian cultivars were homozygous for *mybpa1*, 35 were heterozygous, and only 5 were homozygous for *MYBPA1* (Table 2, Table S10). In contrast, only 3 of 14 Asian wild accessions were homozygous for *mybpa1*. The *Fst* value for Asian pears was 0.18, indicating strong positive selection for *mybpa1* during Asian pear domestication, presumably due to the selection for reduced tannin levels.

**Table 2:** *MYBPA1* genotypes in pear cultivars and wild accessions

Origin	Species	N	MYBAP1	heterozygous	mybap1	a allele frequency
Asia_cultivated	*P. bretshneideri* Rehd	36		1	35	98.61%
	*P.pyrifolia* Nakai	161	5	26	130	88.82%
	*P.ussuriensis* Max	28		2	26	96.43%
	*P. sinkiangensis* Yü	25		6	19	88.00%
Asia_wild	*P. betulifolia* Bunge	7	5	1	1	21.43%
	*P. calleryana Decne*	5	4		1	20%
	*P. pashia*	2		1	1	75.00%
Europe_cultivated	*P. communis* L	49			49	100%
Europe_wild	*P. cordata* Desv	2			2	100%
	*P. cossonii* Rehder	2			2	100%
	*P. spinosa* Forssk	2			2	100%
	*P. mamorensis* Trab	1			1	100%
	*P. nivalis* Jacq	1			1	100%
	*P. pyraster (L.) Burgsd*	2			2	100%
	*P. salicifolia* Pall	2			2	100%
Total		325	14	37	274	90.00%

Interestingly, all 49 European cultivated pears and 12 European wild pears genotyped were homozygous for *mybpa1* (Table 2, Table S10), thus suggesting an independent domestication process in European pears. These results demonstrate that the 57-bp insert in the *MYBPA1* promoter reduces *MYBPA1* expression and PA content, thereby playing a critical role in pear domestication.

## Discussion

This study showed that mutations in the *MYB* genes identified within selective sweeps are instrumental in the domestication of bitterness in apples and pears. These *MYB* genes function as positive regulators of the biosynthesis of PAs, which cause bitterness in these fruits. These findings enhance our understanding of the genetic mechanisms underlying bitterness domestication, as previous research has primarily examined the role of bHLH TFs in regulating bitterness in other crops such as almonds (bitterness due to amygdalin, a cyanogenic diglucoside) (Sánchez-Pérez et al. 2019) and cucurbits (bitterness due to cucurbitacin, a triterpenoid compound) (Shang et al. 2014; Zhou et al. 2016).

Previous studies have demonstrated that multiple *MYB* genes (*MdMYB9, MdMYB11,* and *MdMYB12*) regulate PA biosynthesis in apples (An et al. 2018, 2015; Wang et al. 2018). However, to date, no genetic variants in these genes were shown to be directly associated with variations in PA levels. The present study identified a TE insertion in the CDS of *MdMYBTT*, a gene closely related to *MdMYB9* (Figs. 1e, f). The TE insertion impairs *MdMYBTT* protein function (Figs. 1–3), is fixed in *M. domestica* (Table 1), and is directly linked to low fruit PA levels (Fig. 4). This finding provides new insights into the genetic mechanisms underlying the domestication of bitter wild apples.

Although *mdmybtt* is nearly fixed in *M. domestica*, substantial variations in PA levels persist within this species (Fig. 4a; Tables S1 and S6; Francini & Sebastiani, 2013; Li et al. 2021), suggesting that other genetic variants also influence PA levels. Indeed, additional *MYB* genes were identified in selective sweeps (Fig. 1e) and may contribute to PA levels in different apple varieties. Further studies are required to confirm their roles.

This study identified three pear *MYB* genes within selective sweeps. One of these genes, named *MYBPA1,* because of its high sequence homology to apple *MdMYBPA1* (Fig. 5d), contains a 57-bp insertion in its promoter region in cultivated pears when compared with that in Asian wild pears. This insertion causes loss of *MYBPA1* expression in cultivated pears (Figs. 5e, f). Although the molecular regulation of pear *MYBPA1* function remains largely unexplored, *MdMYBPA1* promotes PA biosynthesis in apples (Wang et al. 2018), indicating a comparable function for pear *MYBPA1*. Our transgenic experiments also confirmed that pear MYBPA1 enhances PA biosynthesis (Figs. 5k-m).

Interestingly, the *MYBPA1* locus was identified in the selective sweep analysis between cultivated and wild Asian pears (Figs. 5c, d), but not between cultivated and wild European pears (Fig. S9). This difference originated from the fixation of the mutant *mybpa1* allele, containing the 57-bp insertion, in both cultivated and wild European pears (Table 2). The lack of genetic diversity at this locus in European pears suggests that wild European pears underwent selection prior to domestication. European wild pears, such as *P. pyraster*, bear significantly larger fruits compared to Asian wild pears, such as *P. betulifolia* and *P. calleryana* (Simionca Mărcășan et al. 2023); this phenomenon is identical to the pre-domestication selection observed in wild apples (*M. sieversii*) and is probably mediated by mammals favoring large fruits (Yao et al. 2015).

This study demonstrated convergent domestication of bitterness in apples and pears. Both species independently evolved the non-bitter trait through mutations in genes regulating the PA biosynthesis pathway. Convergent domestication represents a well-established phenomenon in plants. For example, the quantitative trait locus *KRN2* in maize and its rice ortholog *OsKRN2* underwent convergent selection. These orthologs encode WD40 proteins that interact with a gene of unknown function (*DUF1644*) to negatively regulate grain number in both crops (Chen et al. 2022). Mutations in the *Bt* gene eliminate the bitterness trait selected during the domestication of cucurbits, including cucumber, melon, and watermelon (Shang et al. 2014; Zhou et al. 2016). In sunflower (Blackman et al. 2010), tomato (Soyk et al. 2017), soybean (Wu et al. 2017), and barley (Comadran et al. 2012), flowering time variation is regulated by the *FLOWERING TIME* (*FT*) gene or its paralogs. Modifications in *FT* include cis-regulatory, protein-coding, and copy number variations, contributing to diverse flowering time phenotypes (Purugganan 2019). These findings in apples and pears contribute to this growing body of evidence, offering new perspectives regarding the convergent domestication of crop traits and highlighting the independent but parallel evolution of non-bitter fruits through similar genetic mechanisms.

In conclusion, this study provides valuable insights into the convergent domestication of bitterness in apples and pears, extending our understanding of domestication mechanisms beyond previous research that focused on bHLH TFs in other crops. Additionally, the variations in genetic diversity and selective sweeps between Asian and European pears support their distinct domestication processes. This demonstrates the combined influence of natural and human-mediated selection in shaping domestication traits. By elucidating the genetic basis of reduced bitterness in apples and pears, this study provides a deeper understanding of how selective pressures influence regulatory genes to shape domesticated traits, providing valuable knowledge for future crop improvement and breeding strategies.

## Methods

### Plant materials

For *Malus*, 459 accessions were used in this study, with a wide range of geographic origins (Fig. S10, Table S5). These accessions included 364 cultivars; 21 M*. sieversii*; 4 M*. sylvestris*; and 70 accessions from other *Malus* species, including *M. micromalus*, *M. asiatica*, *M. prunifolia, M. floribunda*, *M. spectabilis*, *M. baccata*, *M. rockii*, *M. mandshurica*, *M. robusta*, *M. hupehensis*, *M. pinyiensis*, *M. sillimensis*, *M. sieboldii*, *M. komarovii*, *M. kansuensis*, *M. toringoides*, *M. xiaojinensis*, and *M. honanensis* (Table S5).

For *Pyrus,* 325 accessions were utilized, encompassing diverse species, including *P. bretschneideri*, *P. pyrifolia*, *P. ussuriensis*, *P. sinkiangensis*, *P. communis*, *P. betulifolia*, *P. calleryana*, *P. pashia*, *P. cordata*, *P. cossonii*, *P. spinosa*, *P. mamorensis*, *P. nivalis*, *P. pyraster*, and *P. salicifolia* (Table S10). Leaf samples from 311 accessions were collected in liquid nitrogen and stored at -80 °C for subsequent analyses.

### Selective sweep analyses

Genome re-sequencing data of 117 apple and 101 pear accessions were downloaded from the NCBI database by using accession numbers SRP075497 (Duan et al. 2017) and PRJNA381668 (Wu et al. 2018), respectively. The re-sequencing data were first cleaned by removing adaptor and low-quality sequences using FastP (v0.23.2) (Chen 2023). The cleaned reads were subsequently mapped to the apple reference genome GDDH13 (v1.1) (Daccord et al. 2017) or *Pyrus pyrifolia* “Yunhong No. 1” (Sun et al. 2023a) by using Burrows-Wheeler Alignment (BWA) tool (v0.7.18) (Li and Durbin 2009). Only uniquely mapped reads were retained for further analyses. The mapped reads were sorted by SAMtools (v1.20) (Danecek et al. 2021), and duplicate reads were identified with Picard (http: //broadinstitute. github.io/picard/).

Variant analyses were conducted following the best practice workflow recommended by Genome Analysis Toolkit (GATK, v4.2.6.1) (McKenna et al. 2010), and the SNPs were initially filtered using the parameters of “QD < 2.0 || MQ < 40.0 || FS > 60.0 || SOR > 3.0 || MQRankSum < − 12.5 || ReadPosRankSum < − 8.0” with the SelectVariants and VariantFiltration packages of GATK. Additionally, variants with missing call frequencies greater than 20% and minor allele frequency less than 5% were excluded using Plink (v1.90b6.5) (Purcell et al. 2007).

To identify selective sweeps, θπ ratio (θπ wild/θπ cultivated) was calculated using vcftools (v0.1.14) (Danecek et al. 2011) with a window size of 50 kb and a window step size of 5 kb. Apple selective sweep analyses were performed in three contrasts: Dom (_scion and _rootstock) vs. Other wild, Dom (_scion and _rootstock) vs. Sie (_K and _X), and Dom (_scion and _rootstock) vs Syl. Pear selective sweep analyses were performed in two contrasts: Asia_cultivar vs. Asia_wild and Europe_cultivar vs. Europe_wild. The top 5% of θπ ratio was used to identify selective sweeps. Genes in the regions of selection sweeps were extracted using bedtools (v2.30.0) (Quinlan and Hall 2010) with the parameters of “-F 0.1”.

### Sequence analysis of genes located in selective sweeps

For apples, gene sequences were downloaded from the reference genomes of four wild apple species (*M. baccata* (Chen et al. 2019), *M. sieversii*, *M. sylvestris*, and *M. fusca* (Mansfeld et al. 2023)) and nine apple cultivars (Hanfu (Qin et al. 2023; Zhang et al. 2019b); Gala (Sun et al. 2020); Honeycrisp (Khan et al. 2022); Antonovka, Fuji, M9, and MM106 (Li et al. 2024b); Golden Delicious and WA38 (Zhang et al. 2024).

For pears, gene sequences were downloaded from the reference genomes of *P. betulifolia* (Shanxiduli), *P. pyrifolia* (Nijisseiki*,* Dangshansuli, and Yunhong No. 1 (Sun et al. 2023a), Cuiguan), and *P. communis* (d’Anjou and Bartlett) reference genome (Chagné et al. 2014; Dong et al. 2020; Gao et al. 2021; Shirasawa et al. 2021; Sun et al. 2023b; Wu et al. 2013; Zhang et al. 2022).

### Phylogenetic tree construction

By using the neighbor-joining method, phylogenetic trees were constructed from the sequences of MYB proteins after alignment of the sequences with the ClustalW tool in conjunction with MEGA version 11.0. Phylogenetic analyses were conducted using a minimum evolution phylogeny test with 1000 bootstrap replicates. The MYB protein sequences were obtained from the NCBI database (https://www.ncbi.nlm.nih.gov/) and the Genome Database for Rosaceae (https://www.rosaceae.org/).

### PA content determination and 4-dimethylaminocinnamaldehyde (DMACA) staining

The absolute content of PA in the fruit flesh of mature apples and pears, transgenic tomato plants, and apple calli were quantified using the HCl-vanillin method (Wang et al. 2022), with three biological repeats involving three technical repeats each. The relative content of PA in 153 apple accessions was determined previously (Lin et al. 2023).

PAs in pear fruit flesh and transgenic apple callus were detected by staining with DMACA solution (DMACA [0.2% w/v] in methanol: 6 M HCl, v/v = 1:1) for 30 min (Li et al., 1996) by using three biological repeats. The intensity of blue coloration indicates PA content in tissues. The darker the color, the higher is the PA content.

### Identification of structure variant (SV) in *MdMYBTT* and pear* MYBPA1*

The 411-bp indel SV among *MdMYBTT* alleles was first identified by aligning gene sequences downloaded from different *Malus* reference genomes. Genome resequencing data and PCR analyses were utilized to genotype the variants across multiple *Malus* accessions. The primers for PCR analysis, namely MdMYBTT/tt-411F and MdMYBTT/tt-411F/R (Table S11), were designed to anneal to the gene sequence flanking the 411-bp insertion site. The PCR analysis was designed to produce a 244-bp DNA fragment for *MdMYBTT* without the insertion and a 655-bp fragment for *mdmybtt* with the insertion. A total of 57 and 389 samples were analyzed using resequencing data and PCR, respectively, with 13 samples analyzed by both methods.

The 57-bp indel SV among pear *MYBPA1* alleles was first identified by aligning gene sequences downloaded from different *Pyrus* reference genomes. Genome resequencing data and PCR analyses were used to genotype the variants across various *Pyrus* accessions. The primers for PCR analysis, namely MYBPA1-57 bp-F and MYBPA1-57 bp-R (Table S11), were designed to anneal to the gene sequence flanking the 57-bp insertion site. The PCR analysis was designed to generate a 550-bp DNA fragment for *MYBPA1* without the insertion, and a 607-bp fragment for *mybpa1* with the insertion. A total of 14 and 311 samples were analyzed by resequencing data and PCR, respectively.

### Transformation of apple callus and tomato plants

For transformation vectors, cDNA of *MdMYBTT*, *mdmybtt*, and pear *MYBPA1* was amplified using PCR primers (Table S11) from *M. robusta* “Balenghaitnag” (W43), *M. domestica* “GL-3,” and *P. betulifolia* (W8), respectively. The primers were designed based on the CDS of MfusH2_15g00466 (Mansfeld et al. 2023), MD15G1051400 GDDH13 v1.1 (Daccord et al. 2017), and Pspp.Chr07.01261.1 (Sun et al. 2023a), respectively, to amplify the full CDS from ATG to the stop codon. The amplified cDNAs were subsequently cloned into the plant transformation vector pSAK778 (Drummond et al. 2009) under the transcriptional control of the CaMV35S promoter. Each pSAK778-derived vector was transferred into *Agrobacterium tumefaciens* GV3101 for transformation of *M. domestica* “Wanglin” callus and “Micro-Tom” tomato following established protocols (An et al. 2017; Sun et al. 2006).

To determine the expression of *MdMYBTT*, *mdmybtt*, and pear *MYBPA1* in transgenic apple callus and tomato plants, quantitative reverse transcription polymerase chain reaction (qRT-PCR) analyses were performed using primers qPCR-MYBTT/mybtt-F and qPCR-MYBTT/mybtt-R; qPCRMYBPA1-F and qPCRMYBPA1-R; and internal control gene primers MdActi-F and MdActi-R, and MdEF1α-F and MdEF1α-R (Table S11) for apple, and SlActin2-F and SlActin2-R, and SLEF1α-F and SLEF1α-R for tomato. The qRT-PCR protocol used was similar to that previously described (Ding et al. 2021) with three biological replicates, each containing three technical replicates. All primers are listed in Table S11.

### Y1H assay

For the Y1H assay, the promoter sequences of *ANR1* (MD10G1311100), *ANR2* (MD05G1335600), *LAR1* (MD16G1048500), *LAR2* (MD13G1046900), *ANS1* (MD06G1071600), and *ANS2* (MD03G1001100) were amplified from “GL-3” using PCR primers described in Table S11. The amplified fragments were inserted into the pAbAi vector. The CDSs of *MdMYBTT* and *mdmybtt* (same as above) were inserted into the pGADT7 vector. The Y1H assay was performed using a Matchmaker™ Gold Yeast One-Hybrid Library Screening System Kit (Clontech, San Francisco, USA) in accordance with the manufacturer’s instructions and previously established conditions (Ding et al. 2021).

### Dual-luciferase reporter assay

The promoter sequences of *ANR1*, *ANR2*, *LAR1*, *LAR2*, *ANS1*, and *ANS2* (same as above) were cloned into the pGreenII 0800-LUC vector to transcriptionally fuse the promoter to the luciferase (LUC) reporter gene. pGreenII 0800-LUC also contains a CaMV35S-Renilla-LUC. The complete CDS of *MdMYBTT* and *mdmybtt* (same as above) was cloned into the pSAK778 vector under the control of the CaMV35S promoter. The two constructs were transferred separately into *A. tumefaciens* GV3101(pSoup) cells, and the cells were then used for the dual-luciferase reporter assay (Ding et al. 2021). Six biological replicates were used per treatment for the luciferase assay.

### EMSA

A 5ʹ-biotinylated oligonucleotide (5ʹ-ACGAAACTTCTGCAACAACAGCGACCGCGCCTACT-3ʹ) was used as the probe. The probe was incubated with the nuclear extract at room temperature for 30 min. The entire reaction mixture was separated on a non-denaturing 0.5 × TBE 6% polyacrylamide gel for 1 h at 60 V at 4 °C and then transferred onto Biodyne® B nylon membranes (Pall Corporation). Signals were visualized with reagents included in the kit and ChemiDoc XRS (Bio-Rad Laboratories, USA).

### Luciferase reporter analysis

LUC reporter assays were conducted as described previously (Wang et al. 2021). The promoter sequences of *MYBPA1* and *mybpa1* were amplified from *P. betulifolia* W8 and *P. pyrifolia* C174 by using PCR primers 0800-MYBPA1-F and 0800-MYBPA1-R (Table S11). The PCR products were transcriptionally fused to the LUC reporter gene in the pGreenII 0800-LUC vector. pGreenII 0800-LUC also contains a CaMV35S-Renilla_LUC. Six biological replicates were utilized per treatment for the luciferase assay.

### Transcriptome sequencing and analysis

For transcriptome analyses, RNA was extracted from pear fruit flesh at 30 and 120 DAFB from *P. betulifolia-1* (Bet_30d/120d), *P. calleryana* (Cal_30d/120d), “Hongxiangsu” (HXS_30/120), and “Dangshansuli” (DSS_30d/120d) using the OMEGA E.Z.N.A. Plant Total RNA Kit (OMEGA, Kentucky, USA) in accordance with the manufacturer’s instructions. For each RNA sample, 5 µg was utilized to construct strand-specific poly-A RNA libraries as described previously (Haile et al. 2017). The libraries were sequenced using the HiSeq2000 sequencing system (Illumina) to generate 20–27 million paired-end reads per library. The reads were aligned to the *P. pyrifolia* “Yunhong No. 1” reference genome (Sun et al. 2023a) using Bowtie2 (Langmead and Salzberg 2012). FPKM (Fragments Per Kilobase of transcript per Million mapped reads) and differential gene expression analyses were conducted following established methods (Trapnell et al. 2010).

### Statistical analysis

Statistical analyses were conducted using analysis of variance (ANOVA) with statistical programs GenStat version 18 (VSNI Ltd., 2011) and SPSS Statistics version 17.0 (SPSS Inc., Chicago, IL, USA). Post-hoc differences between mean values were determined using Fisher’s least significant difference test (Hayter 1986) at the 5% significance level.

## Supplementary Information

Supplementary Material 1. Supplemental Figure S1. A Venn diagram was constructed using the MYB genes identified within the selective sweeps of three distinct comparisons.

Supplementary Material 2. Supplemental Figure S2. MD15G1051400 gene structure variation analysis.

Supplementary Material 3. Supplemental Figure S3. The 411-bp TE inserted into mdmybtt demonstrates high sequence homology to many other sequences in the apple genome, as shown by a BLAST search.

Supplementary Material 4. Supplemental Figure S4. Structural variation and expression analysis of MD14G1234500 and MD14G1234600.

Supplementary Material 4. Supplemental Figure S4. Structural variation and expression analysis of MD14G1234500 and MD14G1234600.

Supplementary Material 6. Supplemental Figure S6. Detection of presence and expression of transgenes in transgenic tomato plants.

Supplementary Material 7. Supplemental Figure S7. MdMYBTT and mdmybtt show no interaction with the promoters of proanthocyanin biosynthesis-related genes.

Supplementary Material 8. Supplemental Figure S8. European pear selective sweep analysis and MYB phylogenetic tree analysis.

Supplementary Material 9. Supplemental Figure S9. Expression and structural variation analysis of Pspp.Chr02.00612.1 and Pspp.Chr02.00613.1.

Supplementary Material 10. Supplemental Figure S10. Geographic distribution of wild and cultivated apple accessions used in this study (a) and their corresponding MdMYBTT genotype (b).

Supplementary Material 11. Supplemental Figure S11. Transcriptome analysis of pear fruit flesh.

Supplementary Material 12. Supplemental Table S1. Absolute proanthocyanin content in apple fruit flesh. Supplemental Table S2. Selective sweeps identified in M. domestica accessions and M. sieversii. Supplemental Table S3. Selective sweeps identified in M. domestica accessions and M. sylvestris. Supplemental Table S4. Selective sweeps identified in M. domestica accessions and Other_wild accessions. Supplemental Table S5. MdMYB-Tannin-Tamer genotyping in 364 apple cultivars and 95 wild varieties. Supplemental Table S6. Relative proanthocyanin content in apple fruit flesh reported by Lin et al. (2023). Supplemental Table S7. Absolute proanthocyanin content in pear fruit flesh. Supplemental Table S8. Selective sweeps identified in comparison between the Asia cultivar and Asia wild pears and genes located inside the sweeps. Supplemental Table S9. Selective sweeps identified in comparison between the European cultivar and European wild pears and genes located inside the sweeps. Supplemental Table S10. MYBPA1 genotyping in 299 pear cultivars and 26 wild varieties. Supplemental Table S11. Sequences of primers used for vector construction, gene cloning, and qRT-PCR.

## Data Availability

The RNA-seg data of the current study were deposited in the Genome Sequence Archive (GSA, https://ngdc.cncb.an.cn/gsa/) of the National Genomics Data Center (NGDC)/China National Center for Bioinformation (CNCB) under the accession number CRA036185. Other data generated and analyzed during this study are included in this published article and its supplementary information files.

## Abbreviations

AbA: Aureobasidin A
DMACA: Dimethylaminocinnamaldehyde
EMSA: Electrophoretic Mobility Shift Assay
FPKM: Fragments Per Kilobase per Million mapped fragments
PA: Proanthocyanidin
Y1H: Yeast one-hybrid
