# Supplementary figures and images for: Convergent domestication of bitter apples and pears by selecting mutations of MYB transcription factors to reduce proanthocyanidin levels
Source: Mol Hortic. 2025 Sep 4;5:51. doi: 10.1186/s43897-025-00173-z (PMC12409940; doi:10.1186/s43897-025-00173-z)

## Slide 1
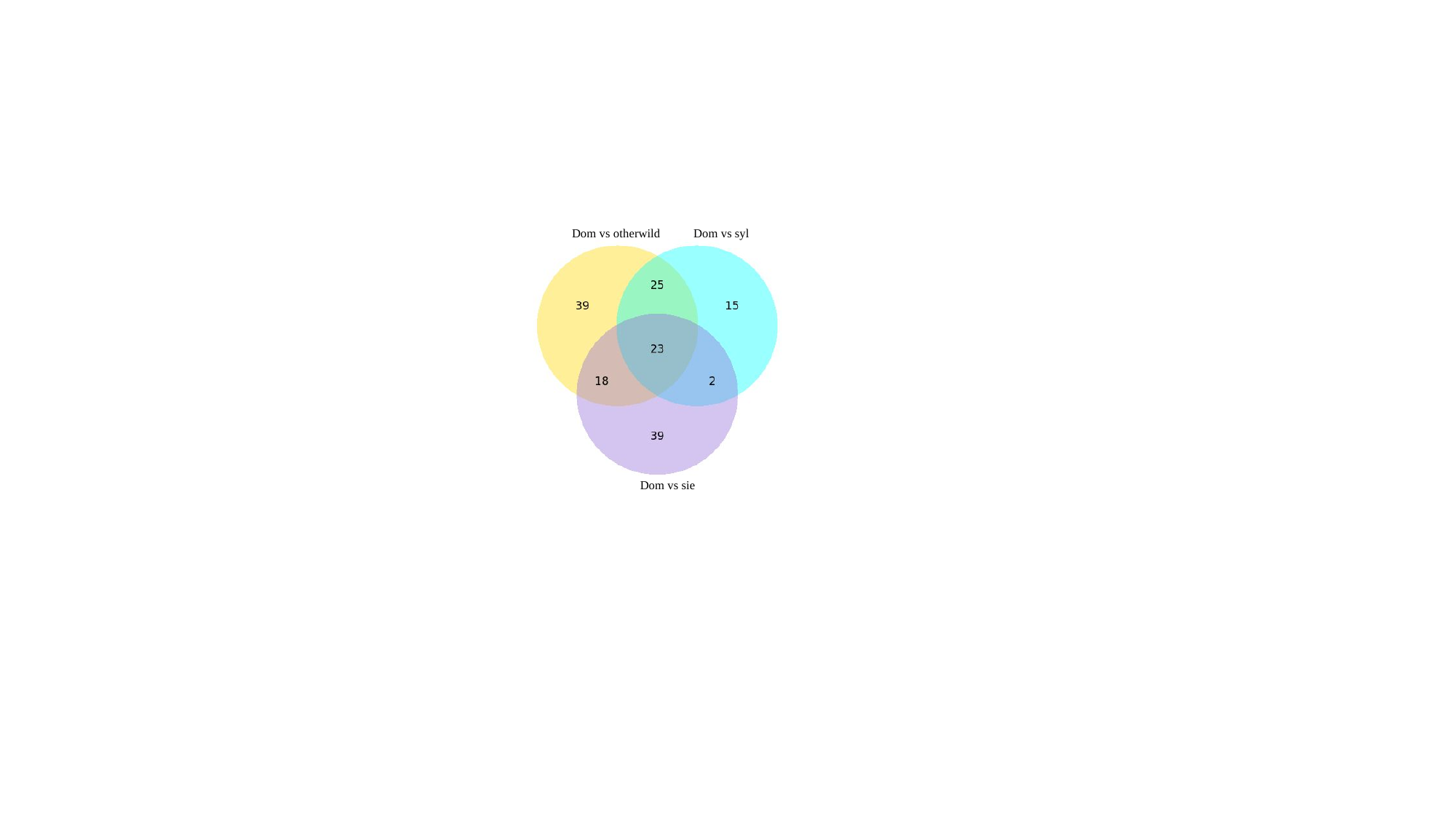

Dom vs syl
Dom vs otherwild
Dom vs sie

Supplement: Supplementary file 1 — Supplementary Material 1. Supplemental Figure S1. A Venn diagram was constructed using the MYB genes identified within the selective sweeps of three distinct comparisons. [file 43897_2025_173_MOESM1_ESM.pptx]

## Slide 1
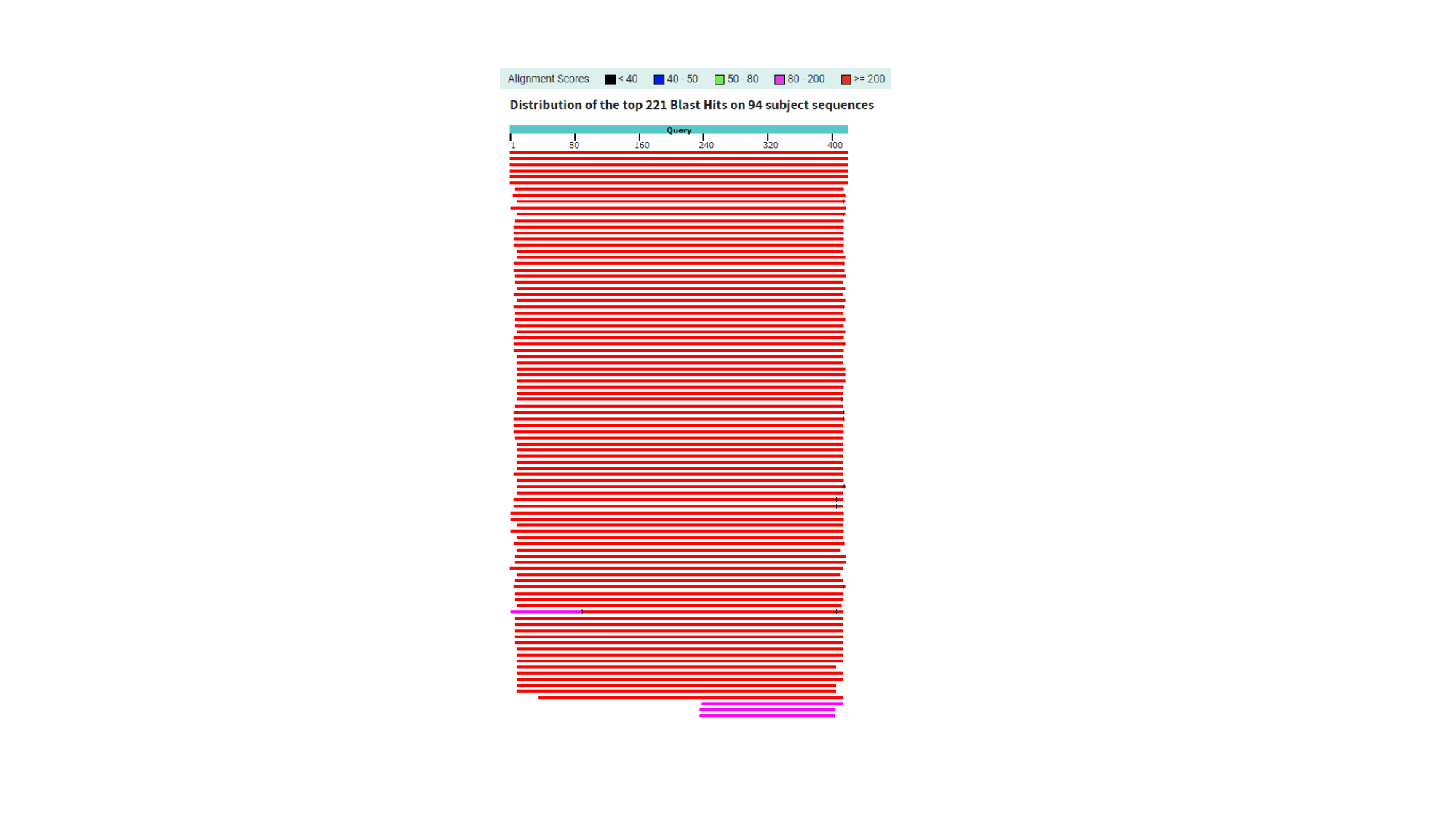

Supplement: Supplementary file 3 — Supplementary Material 3. Supplemental Figure S3. The 411-bp TE inserted into mdmybtt demonstrates high sequence homology to many other sequences in the apple genome, as shown by a BLAST search. [file 43897_2025_173_MOESM3_ESM.pptx]

## Slide 1
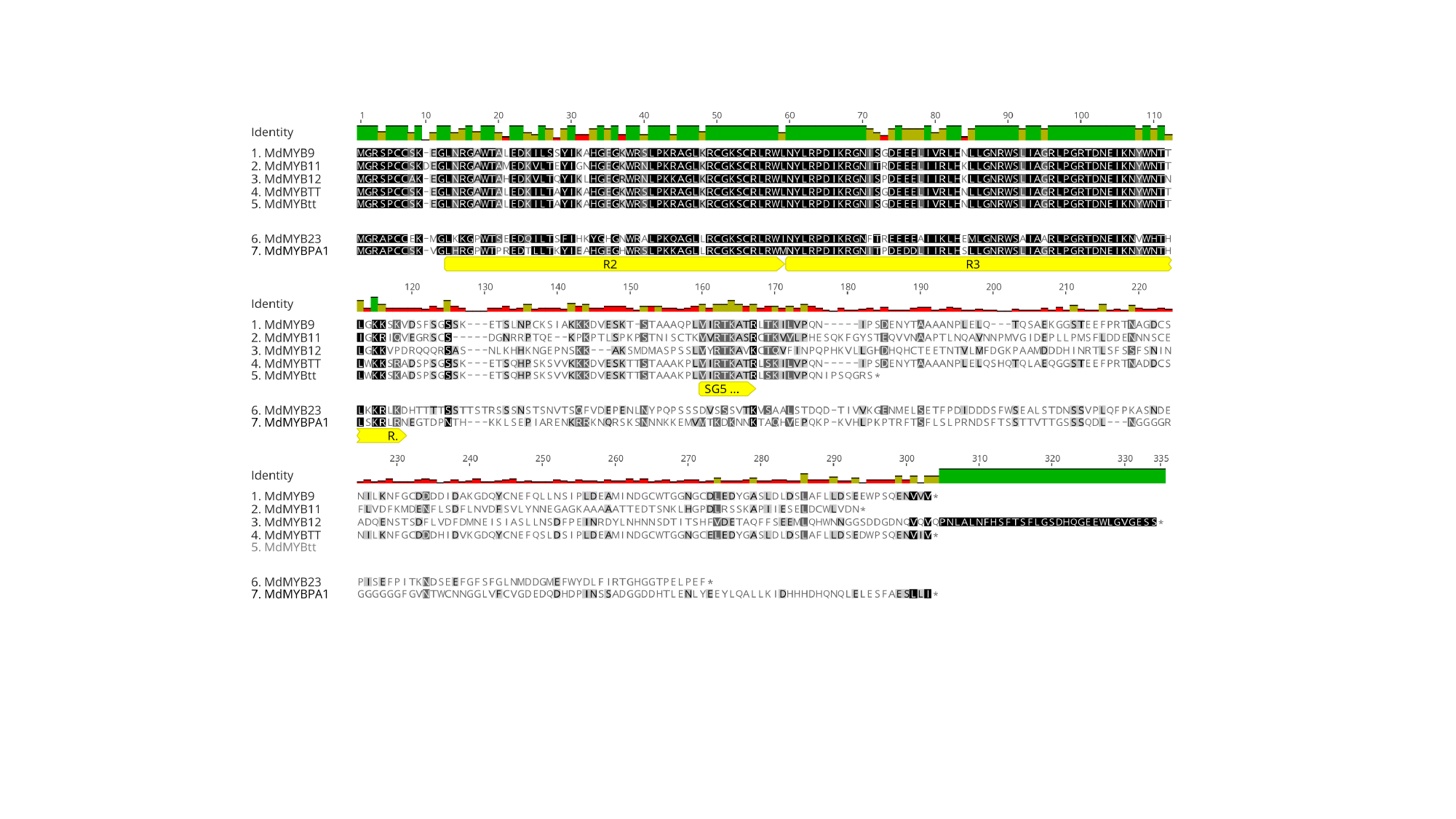

Supplement: Supplementary file 5 — Supplementary Material 4. Supplemental Figure S4. Structural variation and expression analysis of MD14G1234500 and MD14G1234600. [file 43897_2025_173_MOESM5_ESM.pptx]

## Slide 1
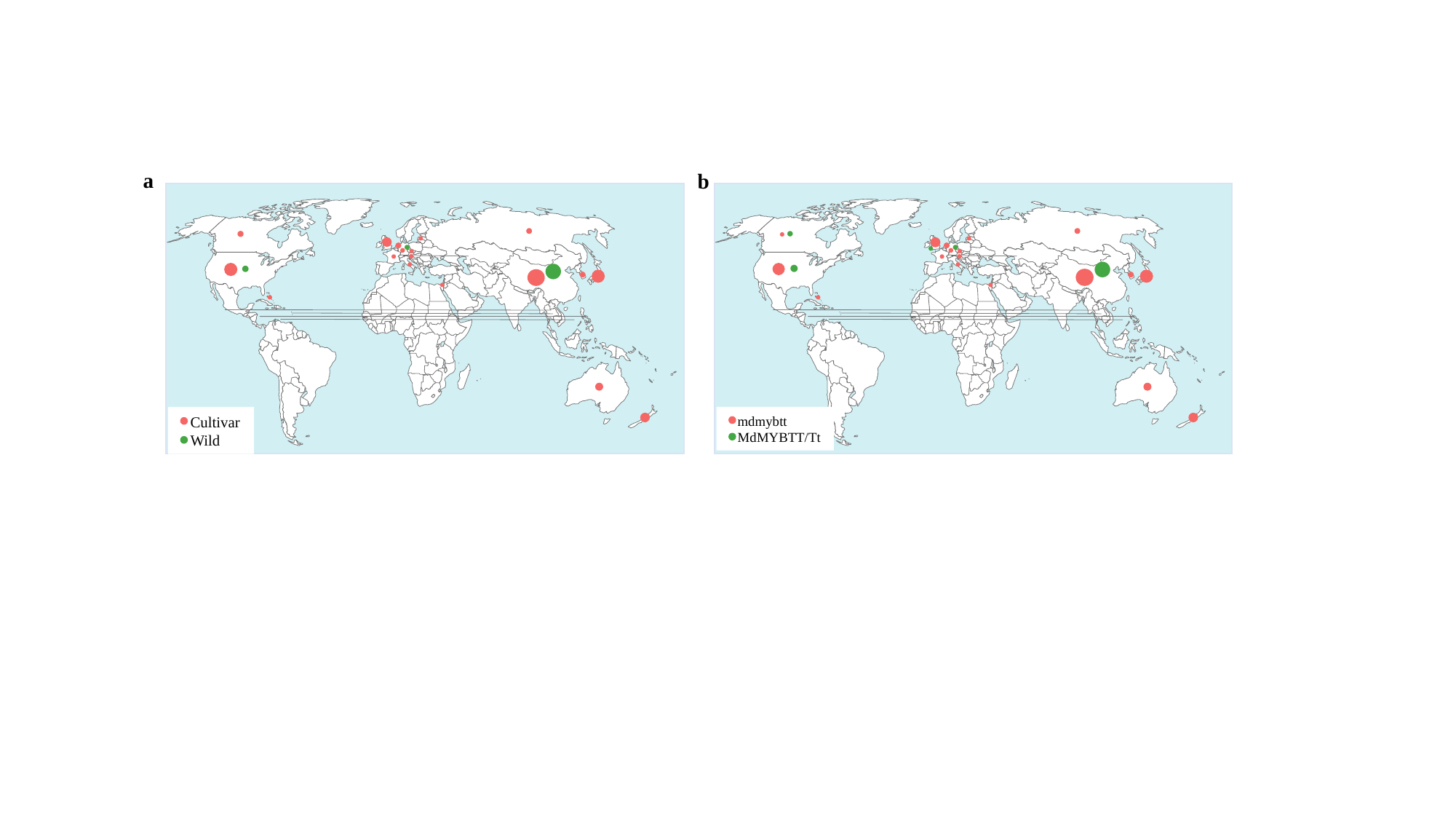

a
b
 Cultivar
 Wild
 mdmybtt
 MdMYBTT/Tt

Supplement: Supplementary file 10 — Supplementary Material 10. Supplemental Figure S10. Geographic distribution of wild and cultivated apple accessions used in this study (a) and their corresponding MdMYBTT genotype (b). [file 43897_2025_173_MOESM10_ESM.pptx]
