# Supplementary material for: Convergent domestication of bitter apples and pears by selecting mutations of MYB transcription factors to reduce proanthocyanidin levels
Source: Mol Hortic. 2025 Sep 4;5:51. doi: 10.1186/s43897-025-00173-z (PMC12409940; doi:10.1186/s43897-025-00173-z)
Supplement: Supplementary file 2 — Supplementary Material 2. Supplemental Figure S2. MD15G1051400 gene structure variation analysis. [file 43897_2025_173_MOESM2_ESM.pptx]

## Slide 1
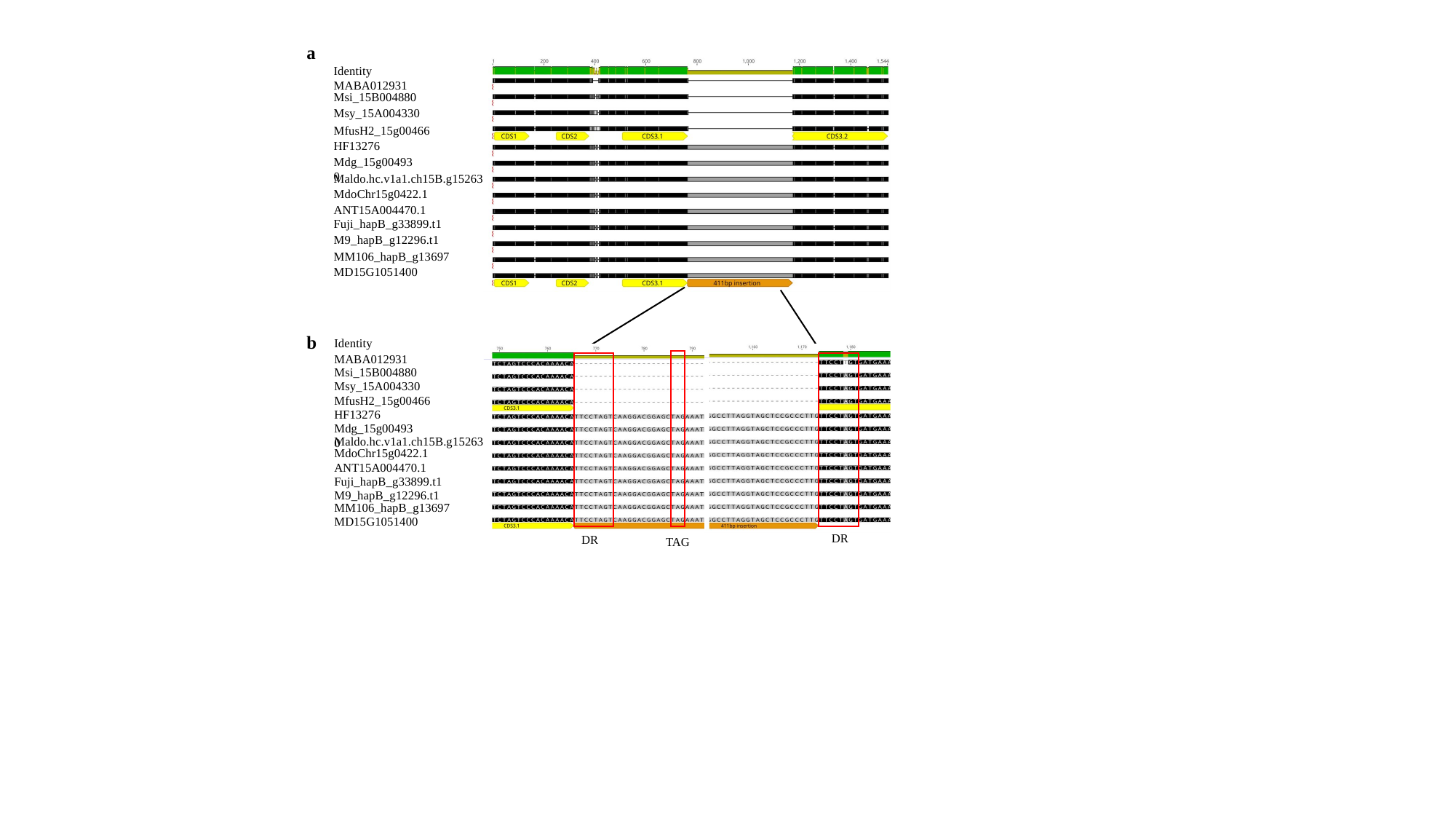

a
Identity
MABA012931
Msi_15B004880
Msy_15A004330
MfusH2_15g00466
HF13276
Mdg_15g004930
Maldo.hc.v1a1.ch15B.g15263
MdoChr15g0422.1
ANT15A004470.1
Fuji_hapB_g33899.t1
M9_hapB_g12296.t1
MM106_hapB_g13697
MD15G1051400
b
Identity
MABA012931
Msi_15B004880
Msy_15A004330
MfusH2_15g00466
HF13276
Mdg_15g004930
Maldo.hc.v1a1.ch15B.g15263
MdoChr15g0422.1
ANT15A004470.1
Fuji_hapB_g33899.t1
M9_hapB_g12296.t1
MM106_hapB_g13697
MD15G1051400
DR
DR
TAG
