# Supplementary material for: Convergent domestication of bitter apples and pears by selecting mutations of MYB transcription factors to reduce proanthocyanidin levels
Source: Mol Hortic. 2025 Sep 4;5:51. doi: 10.1186/s43897-025-00173-z (PMC12409940; doi:10.1186/s43897-025-00173-z)
Supplement: Supplementary file 4 — Supplementary Material 4. Supplemental Figure S4. Structural variation and expression analysis of MD14G1234500 and MD14G1234600. [file 43897_2025_173_MOESM4_ESM.pptx]

## Slide 1
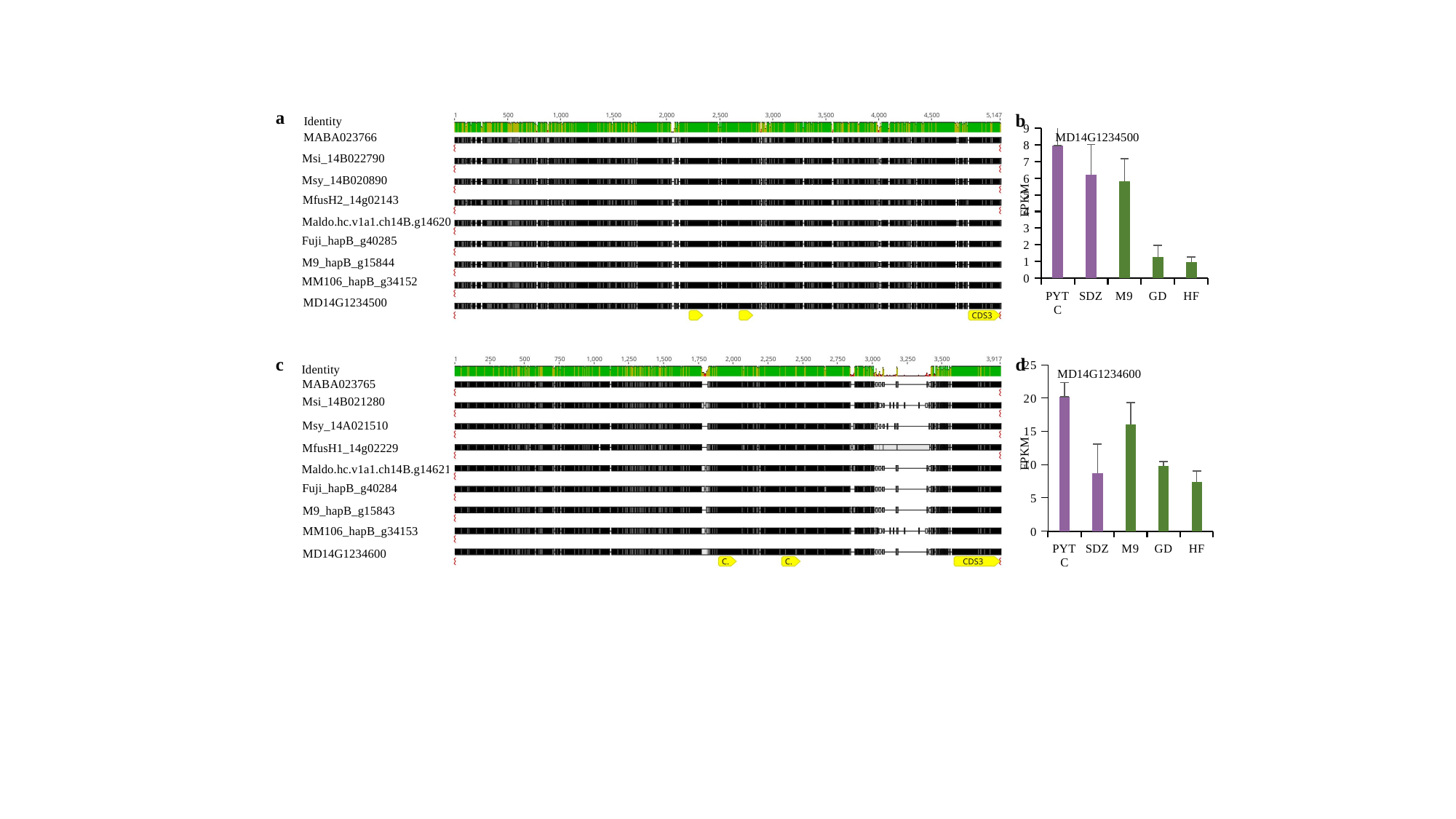

a
b
Identity
MABA023766
Msi_14B022790
Msy_14B020890
MfusH2_14g02143
Maldo.hc.v1a1.ch14B.g14620
Fuji_hapB_g40285
M9_hapB_g15844
MM106_hapB_g34152
MD14G1234500
### Chart
| Category | |
|---|---|
| PYTC | 7.967819087503043 |
| SDZ | 6.2203841563888735 |
| M9 | 5.821392920183176 |
| GD | 1.2802918420712954 |
| HF | 0.9565576798096016 |FPKM
MD14G1234500
c
d
### Chart
| Category | |
|---|---|
| PYTC | 20.21801071221 |
| SDZ | 8.685766376863215 |
| M9 | 16.07236994517167 |
| GD | 9.773674131526178 |
| HF | 7.397227067189447 |FPKM
Identity
MABA023765
Msi_14B021280
Msy_14A021510
MfusH1_14g02229
Maldo.hc.v1a1.ch14B.g14621
Fuji_hapB_g40284
M9_hapB_g15843
MM106_hapB_g34153
MD14G1234600
MD14G1234600
