# Supplementary material for: Convergent domestication of bitter apples and pears by selecting mutations of MYB transcription factors to reduce proanthocyanidin levels
Source: Mol Hortic. 2025 Sep 4;5:51. doi: 10.1186/s43897-025-00173-z (PMC12409940; doi:10.1186/s43897-025-00173-z)
Supplement: Supplementary file 6 — Supplementary Material 6. Supplemental Figure S6. Detection of presence and expression of transgenes in transgenic tomato plants. [file 43897_2025_173_MOESM6_ESM.pptx]

## Slide 1
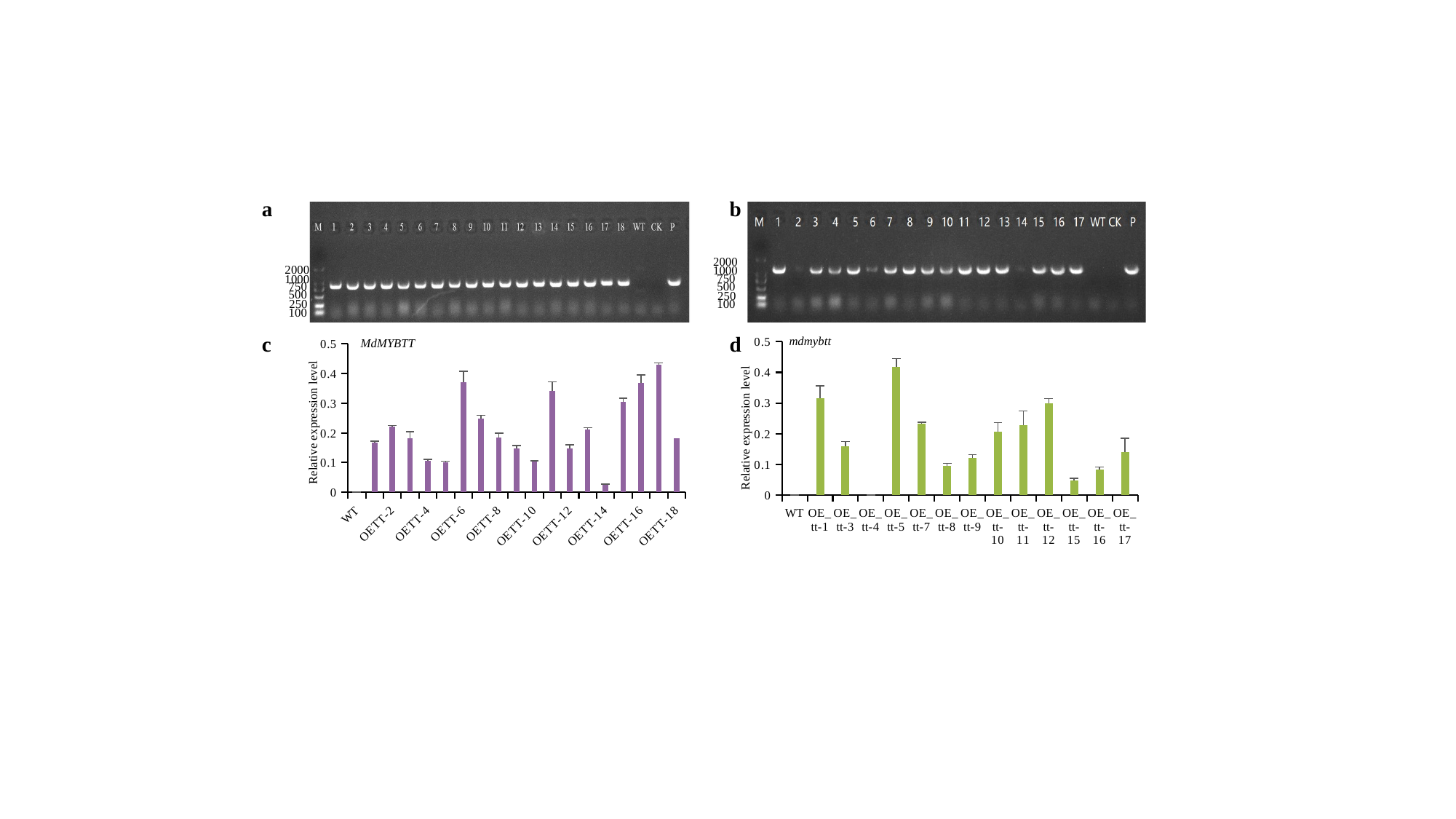

a
b
2000
1000
750
500
250
100
2000
1000
750
500
250
100
MdMYBTT
### Chart
| Category | |
|---|---|
| WT | 0.00012742369509030518 |
| OETT-1 | 0.16698552972246025 |
| OETT-2 | 0.22128047321847857 |
| OETT-3 | 0.18188822200986596 |
| OETT-4 | 0.10565769116161927 |
| OETT-5 | 0.10184298742973247 |
| OETT-6 | 0.370673918073661 |
| OETT-7 | 0.2493338538832237 |
| OETT-8 | 0.1838236918560471 |
| OETT-9 | 0.14762836648066674 |
| OETT-10 | 0.1036096597115473 |
| OETT-11 | 0.34051930914349454 |
| OETT-12 | 0.14737457955466327 |
| OETT-13 | 0.21097103814254645 |
| OETT-14 | 0.02445920987489184 |
| OETT-15 | 0.3050289523977186 |
| OETT-16 | 0.36931264513625267 |
| OETT-17 | 0.4302308524252624 |
| OETT-18 | 0.18187263209147012 |Relative expression level
mdmybtt
### Chart
| Category | |
|---|---|
| WT | 0.00013238059805715879 |
| OE_tt-1 | 0.315805715799973 |
| OE_tt-3 | 0.15998355265624034 |
| OE_tt-4 | 2.0429627630437884e-05 |
| OE_tt-5 | 0.41837217179441294 |
| OE_tt-7 | 0.23289515621372642 |
| OE_tt-8 | 0.0943172133192967 |
| OE_tt-9 | 0.12045132725119773 |
| OE_tt-10 | 0.20650971268247809 |
| OE_tt-11 | 0.22855048380990395 |
| OE_tt-12 | 0.29878205827756155 |
| OE_tt-15 | 0.0479043364197705 |
| OE_tt-16 | 0.08265379431742013 |
| OE_tt-17 | 0.14086394263057625 |Relative expression level
c
d
