# Supplementary material for: Convergent domestication of bitter apples and pears by selecting mutations of MYB transcription factors to reduce proanthocyanidin levels
Source: Mol Hortic. 2025 Sep 4;5:51. doi: 10.1186/s43897-025-00173-z (PMC12409940; doi:10.1186/s43897-025-00173-z)
Supplement: Supplementary file 7 — Supplementary Material 7. Supplemental Figure S7. MdMYBTT and mdmybtt show no interaction with the promoters of proanthocyanin biosynthesis-related genes. [file 43897_2025_173_MOESM7_ESM.pptx]

## Slide 1
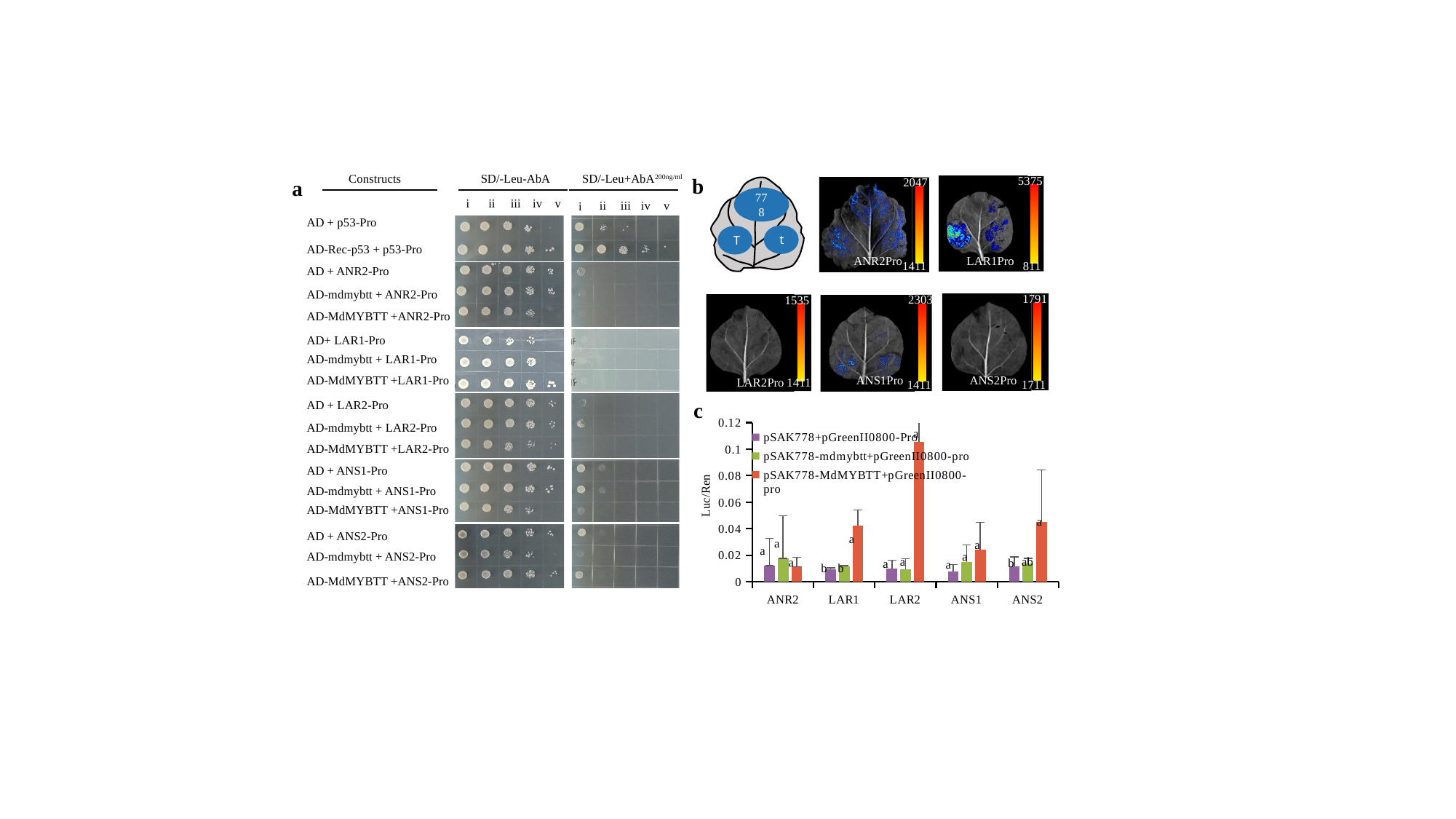

Constructs
SD/-Leu-AbA
SD/-Leu+AbA200ng/ml
b
5375
LAR1Pro
811
a
2047
ANR2Pro
1411
778
t
T
i
ii
iii
iv
v
v
iii
iv
ii
i
AD + p53-Pro
AD-Rec-p53 + p53-Pro
AD + ANR2-Pro
AD-mdmybtt + ANR2-Pro
ANR1Pro
1791
ANS2Pro
1711
2303
ANS1Pro
1411
ANS1Pro
1535
1411
LAR2Pro
AD-MdMYBTT +ANR2-Pro
AD+ LAR1-Pro
AD-mdmybtt + LAR1-Pro
AD-MdMYBTT +LAR1-Pro
### Chart
| Category | pSAK778+pGreenII0800-Pro | pSAK778-mdmybtt+pGreenII0800-pro | pSAK778-MdMYBTT+pGreenII0800-pro |
|---|---|---|---|
| ANR2 | 0.012079778283181136 | 0.017724208507989905 | 0.011386049702841223 |
| LAR1 | 0.00955054466686098 | 0.011717230412533088 | 0.04203306581689107 |
| LAR2 | 0.009722600345871584 | 0.009622791149229253 | 0.10522799059012192 |
| ANS1 | 0.007943093114806022 | 0.014590844826763498 | 0.024356011411472065 |
| ANS2 | 0.011445774940554229 | 0.013916834579519713 | 0.04486848342327674 |a
Luc/Ren
a
a
a
a
a
a
a
ab
a
b
a
a
b
b
c
AD + LAR2-Pro
AD-mdmybtt + LAR2-Pro
AD-MdMYBTT +LAR2-Pro
AD + ANS1-Pro
AD-mdmybtt + ANS1-Pro
AD-MdMYBTT +ANS1-Pro
AD + ANS2-Pro
AD-mdmybtt + ANS2-Pro
AD-MdMYBTT +ANS2-Pro
