# Supplementary material for: Convergent domestication of bitter apples and pears by selecting mutations of MYB transcription factors to reduce proanthocyanidin levels
Source: Mol Hortic. 2025 Sep 4;5:51. doi: 10.1186/s43897-025-00173-z (PMC12409940; doi:10.1186/s43897-025-00173-z)
Supplement: Supplementary file 8 — Supplementary Material 8. Supplemental Figure S8. European pear selective sweep analysis and MYB phylogenetic tree analysis. [file 43897_2025_173_MOESM8_ESM.pptx]

## Slide 1
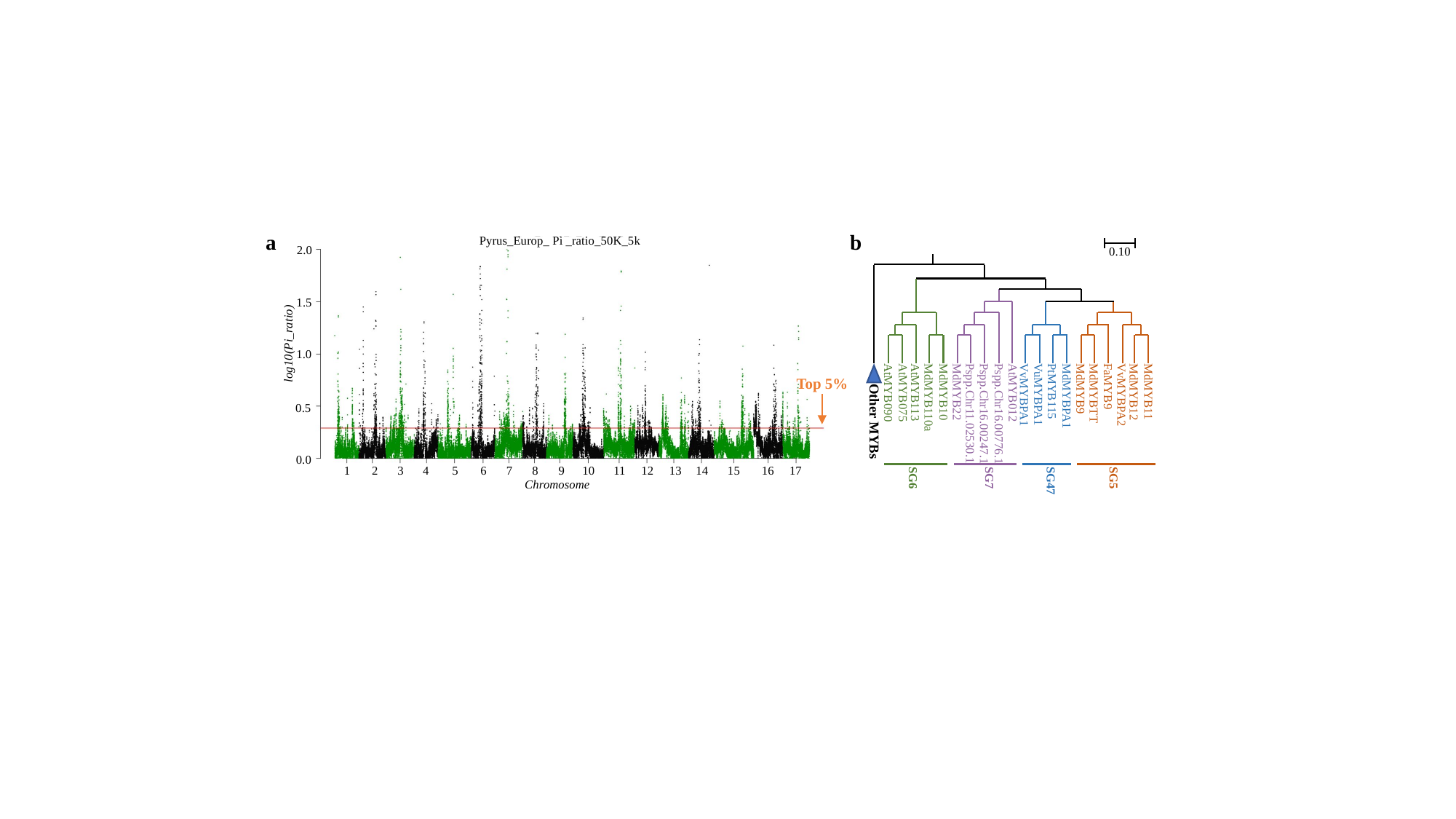

a
b
Pyrus_Europ_ Pi _ratio_50K_5k
Top 5%
2.0
1.5
log10(Pi_ratio)
1.0
0.5
0.0
1
2
3
4
5
6
7
8
9
10
11
12
13
14
15
16
17
Chromosome
0.10
MdMYB11
MdMYB12
VvMYBPA2
SG5
FaMYB9
MdMYBTT
MdMYB9
MdMYBPA1
PtMYB115
SG47
VuMYBPA1
VvMYBPA1
AtMYB012
Pspp.Chr16.00776.1
SG7
Pspp.Chr16.00247.1
Pspp.Chr11.02530.1
MdMYB22
MdMYB10
MdMYB110a
AtMYB113
SG6
AtMYB075
AtMYB090
Other MYBs
