# Supplementary material for: Convergent domestication of bitter apples and pears by selecting mutations of MYB transcription factors to reduce proanthocyanidin levels
Source: Mol Hortic. 2025 Sep 4;5:51. doi: 10.1186/s43897-025-00173-z (PMC12409940; doi:10.1186/s43897-025-00173-z)
Supplement: Supplementary file 9 — Supplementary Material 9. Supplemental Figure S9. Expression and structural variation analysis of Pspp.Chr02.00612.1 and Pspp.Chr02.00613.1. [file 43897_2025_173_MOESM9_ESM.pptx]

## Slide 1
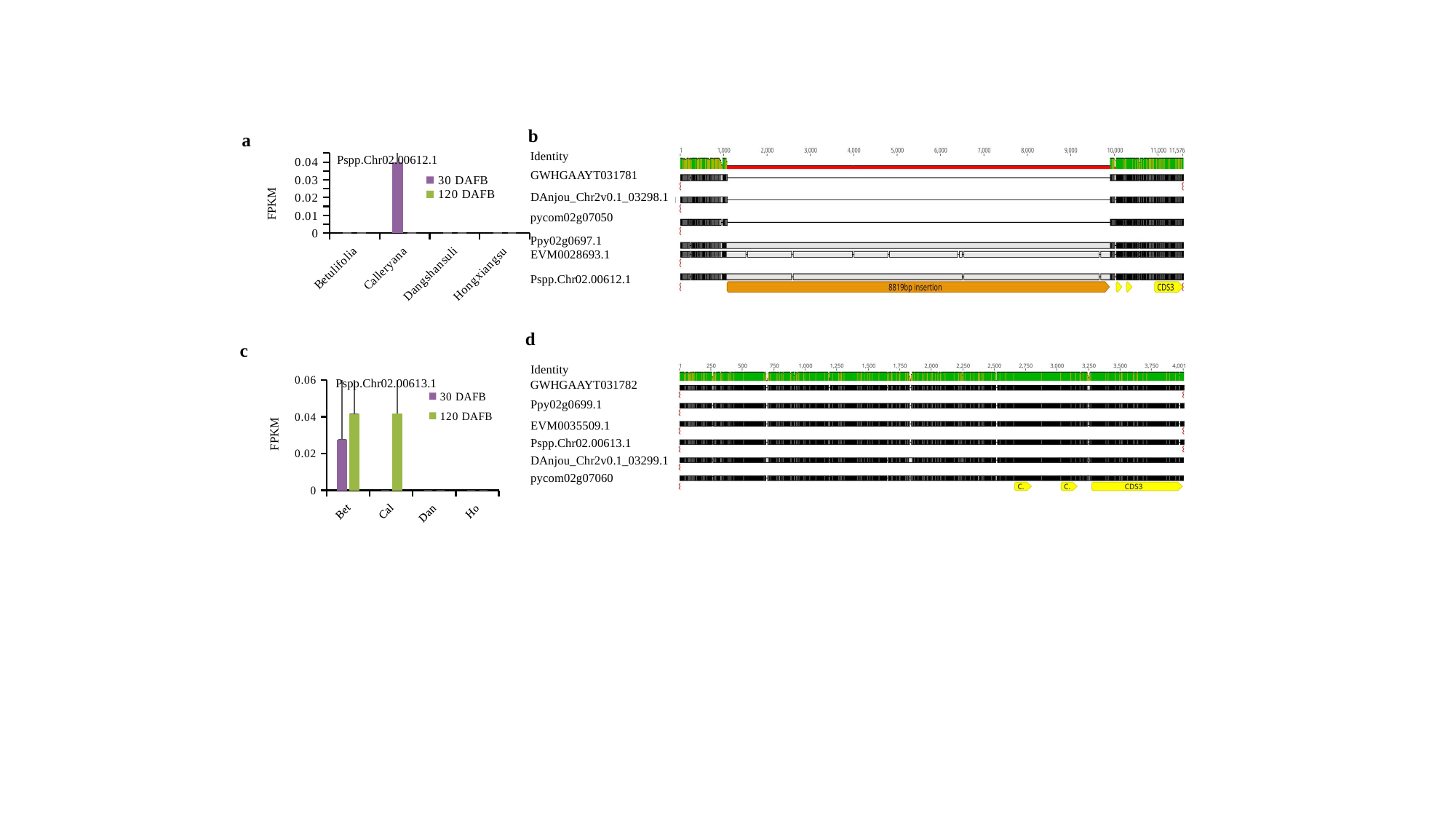

b
a
Identity
Pspp.Chr02.00612.1
### Chart
| Category | 30 DAFB | 120 DAFB |
|---|---|---|
| Betulifolia | 0.0 | 0.0 |
| Calleryana | 0.03999811879653 | 0.0 |
| Dangshansuli | 0.0 | 0.0 |
| Hongxiangsu | 0.0 | 0.0 |GWHGAAYT031781
DAnjou_Chr2v0.1_03298.1
FPKM
pycom02g07050
Ppy02g0697.1
EVM0028693.1
Pspp.Chr02.00612.1
d
c
### Chart
| Category | 30 DAFB | 120 DAFB |
|---|---|---|
| Betulifolia | 0.0277247393449073 | 0.04159420060661647 |
| Calleryana | 0.0 | 0.04159420060661647 |
| Dangshansuli | 0.0 | 0.0 |
| Hongxiangsu | 0.0 | 0.0 |Identity
Pspp.Chr02.00613.1
GWHGAAYT031782
Ppy02g0699.1
EVM0035509.1
FPKM
Pspp.Chr02.00613.1
DAnjou_Chr2v0.1_03299.1
pycom02g07060
