# Supplementary material for: Convergent domestication of bitter apples and pears by selecting mutations of MYB transcription factors to reduce proanthocyanidin levels
Source: Mol Hortic. 2025 Sep 4;5:51. doi: 10.1186/s43897-025-00173-z (PMC12409940; doi:10.1186/s43897-025-00173-z)
Supplement: Supplementary file 11 — Supplementary Material 11. Supplemental Figure S11. Transcriptome analysis of pear fruit flesh. [file 43897_2025_173_MOESM11_ESM.pptx]

## Slide 1
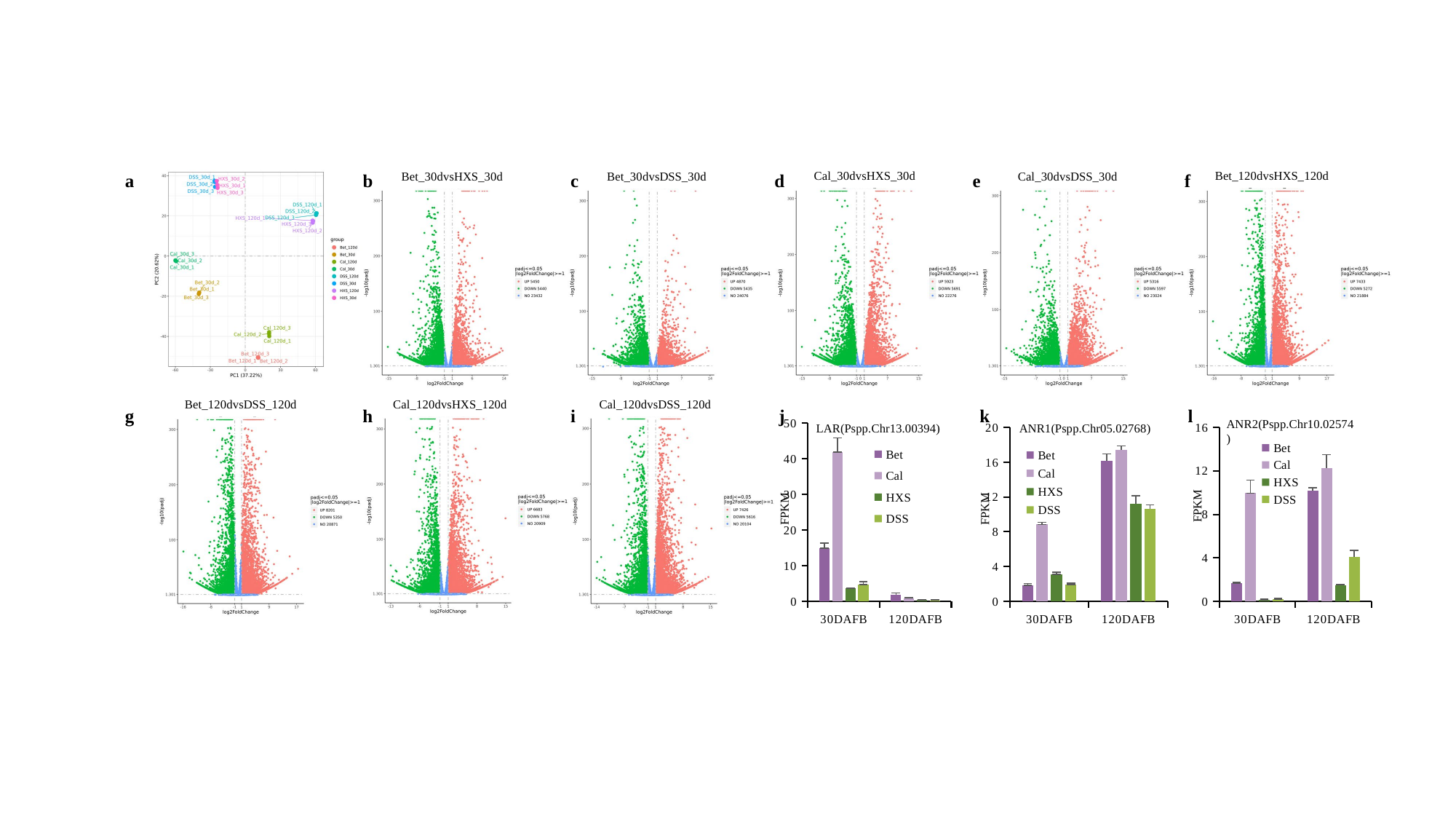

Bet_120dvsHXS_120d
Cal_30dvsHXS_30d
Cal_30dvsDSS_30d
Bet_30dvsHXS_30d
Bet_30dvsDSS_30d
a
b
c
d
e
f
Cal_120dvsHXS_120d
Cal_120dvsDSS_120d
Bet_120dvsDSS_120d
g
h
i
j
k
l
### Chart
| Category | Bet | Cal | HXS | DSS |
|---|---|---|---|---|
| 30DAFB | 14.843120460124235 | 41.771074203755866 | 3.6292336825572 | 4.693959041996307 |
| 120DAFB | 1.79610833167024 | 0.8873086318609 | 0.3165992462615337 | 0.2728235929668487 |ANR2(Pspp.Chr10.02574)
### Chart
| Category | Bet | Cal | HXS | DSS |
|---|---|---|---|---|
| 30DAFB | 1.6692457973672068 | 9.949182871355568 | 0.1311786877865306 | 0.22137144274783602 |
| 120DAFB | 10.193316495120163 | 12.232724047187332 | 1.4666894495216065 | 4.115802457249097 |LAR(Pspp.Chr13.00394)
### Chart
| Category | Bet | Cal | HXS | DSS |
|---|---|---|---|---|
| 30DAFB | 1.85879553758292 | 8.8406754498762 | 3.11784825044478 | 1.91562463764697 |
| 120DAFB | 16.1203240600796 | 17.385970755040166 | 11.174399885262668 | 10.659110015773132 |ANR1(Pspp.Chr05.02768)
FPKM
FPKM
FPKM
